# Strong field spectroscopy of many-body interactions in solids

**DOI:** 10.1038/s41467-026-78716-1

**Published:** 2026-10-20

**Authors:** Viacheslav Korolev, Thomas Lettau, Vipin Krishna, Alexander Croy, Martin Thümmler, Michael Zürch, Christian Spielmann, Maria Wächtler, Ulf Peschel, Stefanie Gräfe, Giancarlo Soavi, Daniil Kartashov

**Affiliations:** 1 Institute of Optics and Quantum Electronics, Friedrich Schiller University Jena, Jena, Germany; 2 Institute of Condensed Matter Theory and Solid State Optics, Friedrich Schiller University Jena, Jena, Germany; 3 Institute of Solid State Physics, Friedrich Schiller University Jena, Jena, Germany; 4 Institute of Physical Chemistry, Friedrich Schiller University Jena, Jena, Germany; 5 Department of Chemistry, University of California at Berkeley, Berkeley, CA USA; 6 Materials Sciences Division, Lawrence Berkeley National Laboratory, Berkeley, CA USA; 7 Helmholtz Institut Jena, Jena, Germany; 8 Abbe Center of Photonics, Jena, Germany; 9 Institute of Physical Chemistry, Kiel University, Kiel, Germany; 10 Fraunhofer Institute for Applied Optics and Precision Engineering, Jena, Germany

**Keywords:** High-harmonic generation, Nonlinear optics

## Abstract

High-order harmonic generation (HHG) in solids offers a promising route to compact ultrafast sources of quantum light, but its efficiency and coherence are limited by ultrafast dephasing of laser-driven electron–hole polarization. Although widely described by a phenomenological coherence time, the microscopic origin of this dephasing has remained unresolved. Here, we combine wavelength-dependent HHG measurements in monolayer and bulk semiconductors with ab initio many-body calculations of electron–phonon scattering and strong-field simulations. We show that the measured wavelength scaling of the HHG yield is quantitatively reproduced only when semiconductor Bloch equations incorporate momentum-dependent dephasing derived from first principles scattering rates. The calculations reveal that strong-field-driven carriers rapidly enter regions of the Brillouin zone with enhanced electron–phonon scattering, giving rise to effective few-femtosecond dephasing. Our results establish a microscopic foundation for dephasing in solid-state HHG and provide a framework for engineering coherence in strongly driven solids for quantum photonic applications.

## Abstract (web summary)

Despite numerous studies on solid-state high-harmonic generation (HHG), there is a lack of microscopic understanding of decoherence effects. Here, the authors study experimentally and by real-time ab-initio simulations the wavelength dependence of HHG in monolayer films, promoting momentum-resolved electron-phonon scattering as the dominant many-body interaction causing ultrafast decoherence.

## Introduction

High-order harmonic generation (HHG) in solids occurs when an intense laser field drives electrons from the valence to the conduction band and accelerates them across the Brillouin zone within a single optical cycle, inducing ultrafast oscillating intraband currents and interband polarization^1–5^. This nonperturbative frequency-conversion process can generate a broad coherent spectrum from the infrared to the extreme-ultraviolet (XUV), enabling tabletop attosecond pulse sources and new modalities of ultrafast spectroscopy^6–16^. In contrast to atomic gases, where a single active electron picture often suffices, HHG in solids is inherently a multiparticle phenomenon: the strong-field excitation creates electron–hole pairs whose coherent polarization radiates high-order harmonics^1–5^. As a result, solid HHG is exquisitely sensitive to many-body interactions that dephase the electron–hole motion. Indeed, the interband recombination mechanism—the solid-state analogue of the atomic three-step model—relies on phase-locked electron and hole trajectories, which are easily disrupted by scattering processes. Such decoherence has no counterpart in gas HHG and must be accounted for in solids: in theory, this is done by introducing a polarization decoherence time on the order of only a few femtoseconds^1–3^. Strikingly, the physical origin of this ultrafast dephasing has remained unclear, and the required dephasing times are far shorter than those known from conventional weak-field nonlinear optics^17–20^. For example, excitonic resonances and low-order nonlinearities in semiconductors typically exhibit coherence times of tens of femtoseconds^17–20^, whereas replicating solid HHG spectra has demanded dephasing times on the order of 2–10fs^1–3^. This discrepancy fueled debate, with some studies suggesting that an ultrashort dephasing time might not be intrinsic but rather an artifact avoidable by considering propagation effects^21^. Overall, a deeper understanding of the microscopic origin and timescale of dephasing in solid HHG has been one of the most important open questions in the field^22,23^, as it directly impacts our ability to model, predict, and harness HHG in novel materials.

Beyond its classical aspects, solid HHG has recently attracted attention as a potential source of quantum light. Experiments in atomic HHG first revealed quantum-optical signatures in high harmonics, sparking the idea that strong-field processes can generate entangled photons despite involving macroscopic photon fluxes^24–32^. Very recently, HHG in semiconductors has been shown to produce nonclassical light: violating multi-mode Cauchy–Schwarz inequalities and exhibiting two-mode squeezing in the emitted harmonics^33^. These observations position solid-state HHG as a promising route to generate bright entangled photon states, bridging ultrafast nonlinear optics with quantum information science. At the same time, any quantum coherence encoded in the emitted harmonics will be fundamentally constrained by the dephasing of the underlying electron–hole polarization during the strong-field interaction. Identifying the microscopic origin of this ultrafast dephasing is therefore important not only for modeling HHG spectra and efficiencies but also for assessing the prospects of solid-state HHG as a source of coherent and quantum light.

Previous studies have shown that the HHG response of solids is highly sensitive to the assumed dephasing time^1–3,34^ and to the carrier population, including under photo-doping conditions where additional carriers enhance scattering^35–37^. In many such treatments, however, the dephasing time is introduced phenomenologically, often as a constant parameter or an empirical momentum-dependent function. This leaves open the question of whether the ultrafast dephasing, required in HHG simulations, can be connected directly to microscopic scattering processes under experimentally relevant strong-field conditions, and to what extent different scattering channels dominate in different excitation regimes.

Here, we address this problem by combining wavelength-dependent HHG measurements on monolayer WS_2_, MoS_2_ and bulk CdSe with ab initio calculations of momentum-resolved scattering rates and strong-field simulations. In our experiments, the total high-harmonic output drops precipitously as the drive wavelength increases, with a steeper efficiency fall-off than in atomic gases^38^. We show that the experimentally observed scaling of the integrated HHG yield with driving wavelength is not reproduced by calculations without dephasing and is highly sensitive to the form of the dephasing term. By introducing a momentum-dependent dephasing time ${T}_{2}$, derived from first-principles electron–phonon scattering rates, we obtain good agreement with the experiment and identify a clear microscopic origin for the ultrafast effective dephasing in the present excitation regime. This momentum-resolved dephasing model unifies the description of ultrafast many-body dynamics in both the perturbative (weak-field) and nonperturbative (strong-field) regimes of solid-state optics, as it converges to long ${T}_{2}$ values in the low-excitation limit and to ultrashort ${T}_{2}$ in extreme driving fields.

## Results

### High harmonic spectra of atomically thin and bulk semiconductors

The experiments are carried out on monolayer WS_2_ and MoS_2_ transition metal dichalcogenides (TMDs) to avoid the influence of linear and nonlinear propagation effects. Further, we compare the results of atomically thin semiconductors to HHG obtained from a bulk polycrystalline CdSe thin film. TMD monolayers were obtained by mechanical exfoliation and transferred onto a c-cut sapphire substrate (see Supplementary Information Section 1). The bulk, polycrystalline, 140 nm thick, wurtzite symmetry CdSe film was prepared directly on the sapphire substrate via chemical bath deposition. We excite the samples with intense (up to 1.5 TW/cm^2^) femtosecond mid-IR pulses at different wavelengths in the range *λ* = 3–5 µm and measure high-order harmonic spectra within the 0.2–1 µm spectral region at a constant laser intensity (see “Methods” and Supplementary Information Section 2). In our analysis, we focus on harmonics with photon energies larger than the electronic bandgap, i.e., those that are generated predominantly by the interband mechanism.

**Fig. 1:**
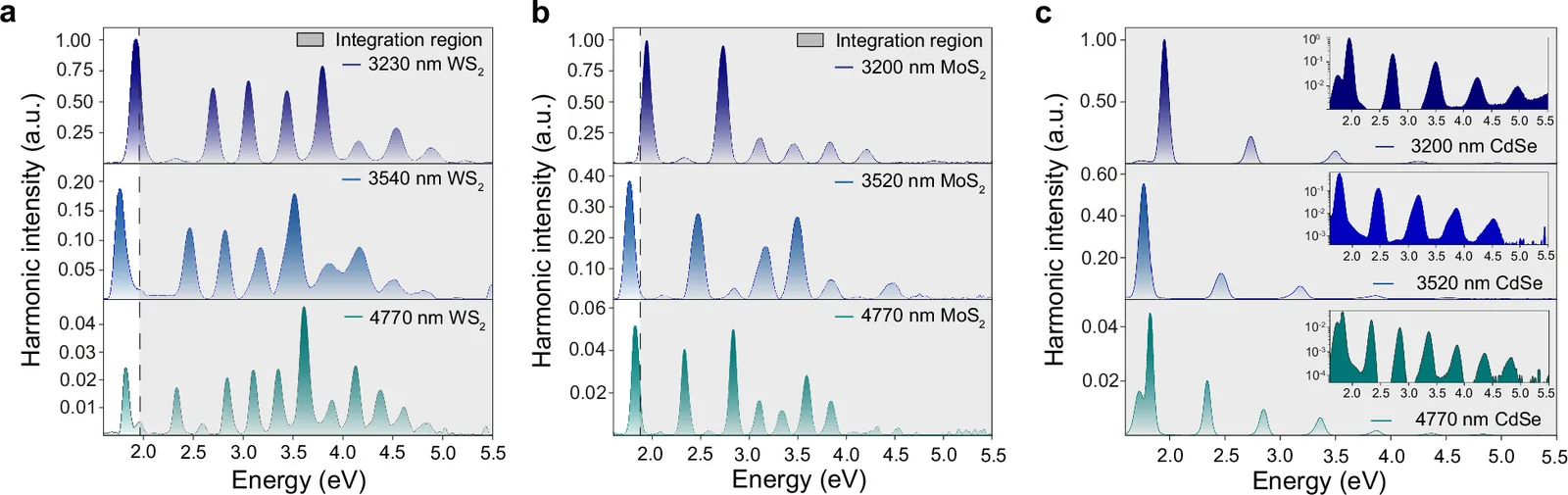
Wavelength dependence in solid-state HHG. HHG spectra on linear scale, corrected to the spectrometer and the detector sensitivity, for three selected laser wavelengths from **a** WS_2_ along the zigzag direction, **b** MoS_2_ along the zigzag direction and **c** CdSe polycrystalline film. The inset figures are the same, but in logarithmic scale for better visibility. The shaded area on all figures marks harmonics with energies of quanta above the bandgap (dashed line) and represents the integration region for the harmonic yield. Note the different *y*-axis range for the different driving wavelength, indicating the reduced harmonic yield for longer driving wavelengths.

Figure 1 shows selected high harmonic spectra obtained from two different TMD monolayers (WS_2_, a, and MoS_2_, b), oriented along the zigzag direction relative to the laser polarization, and from the CdSe film (c) at a peak intensity of approximately 1.5 TW/cm^2^. In TMD monolayers, for symmetry reasons, both even and odd harmonics are generated up to 5 eV, and the spectra exhibit a clear plateau behavior with a cut-off near 4 eV. In contrast, harmonic spectra measured from the bulk CdSe contain only odd orders, and the intensities exponentially decay with increasing harmonic order. This can be explained by strong re-absorption of photons with energies above the bandgap (1.64 eV) of the material^39^. Note that for a thickness of 140 nm, CdSe film dispersion effects like phase and group velocity mismatch are negligible in the entire spectral range of harmonic measurements.

### Experimental wavelength dependence

**Fig. 2:**
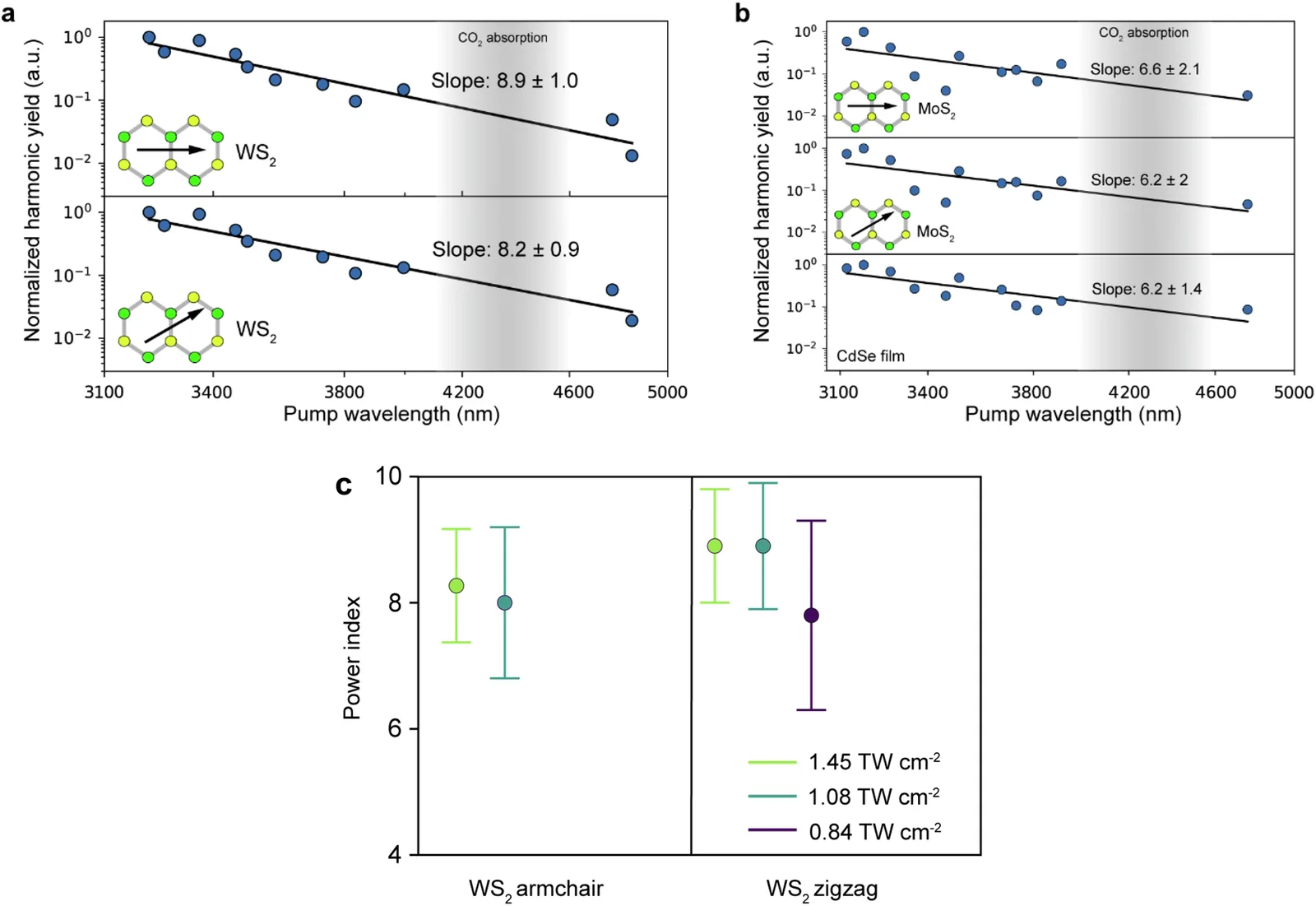
Wavelength dependence of the high harmonic yield. **a** Normalised harmonic yield (blue dots) as a function of the pump wavelength plotted in a double logarithmic scale from a monolayer WS_2_ along the zigzag (top figure) and the armchair (bottom figure) directions. **b** The same for MoS_2_, top figure along the zigzag direction, middle along the armchair and bottom for CdSe film. The values represent the integrated harmonic yield in the spectral region above the bandgap (marked in Fig. 1). The black line shows a linear fit and the absolute values of the slopes are shown. The shaded grey area represents the CO_2_ absorption region, where measurements are not possible under ambient conditions. The laser intensity was fixed to 1.45 TW/cm^2^. **c** Experimentally measured dependence of the power index in the wavelength-dependent decay of the harmonic yield on the driving laser intensity, for two different crystal orientations of WS_*2*_. The error bars indicate slope fitting error.

Figure 2 displays the experimentally retrieved wavelength dependence of the integral harmonic yield, measured at different pump laser wavelengths but at a fixed laser intensity of 1.45 TW/cm^2^ (for details on the intensity calibration, see Supplementary Information Section 2.2). In our analysis, we focus on harmonics with photon energies larger than the electronic bandgap, i.e., those generated predominantly by the interband mechanism. We define the HHG yield as the detector- and grating-corrected spectral intensity integrated, in the frequency domain, over this spectral range within the experimentally accessible window indicated by the shaded area in Fig. 1. The same integration window is used in the comparison with numerical simulations. The presentation on a double logarithmic scale allows convenient fitting of a power-law decay λ^-x^ with a linear function, characterizing the power index × of its decay by the value of the slope. For monolayer WS_2_ (Fig. 2a), we find values of the power index of −8.9 ± 1 and 8.2 ± 0.9 along the zigzag and armchair directions, respectively. Similarly, Fig. 2b shows the wavelength dependence of the total HHG yield from a MoS_2_ monolayer along the zigzag and armchair directions and from the CdSe film, with retrieved indices −6.6 ± 2 (MoS_2_ zigzag) and −6.2 ± 1.4 (both MoS_2_ (armchair) and CdSe). The power indices are only weakly dependent on the laser intensity, remaining almost unaffected (<10%) when the latter is changed by more than 70% (Fig. 2c). Note that in all cases, the harmonic yield does not decay monotonically but shows an oscillatory behavior instead. Similar oscillations have been numerically found before and were assigned to quantum path interferences^34^. In general, the experimentally retrieved power index is significantly larger than the theoretically proposed ${\lambda }^{-4}$ dependence expected assuming an infinite dephasing time (no dephasing processes)^34^.

### Benchmark against no dephasing or constant dephasing models

**Fig. 3:**
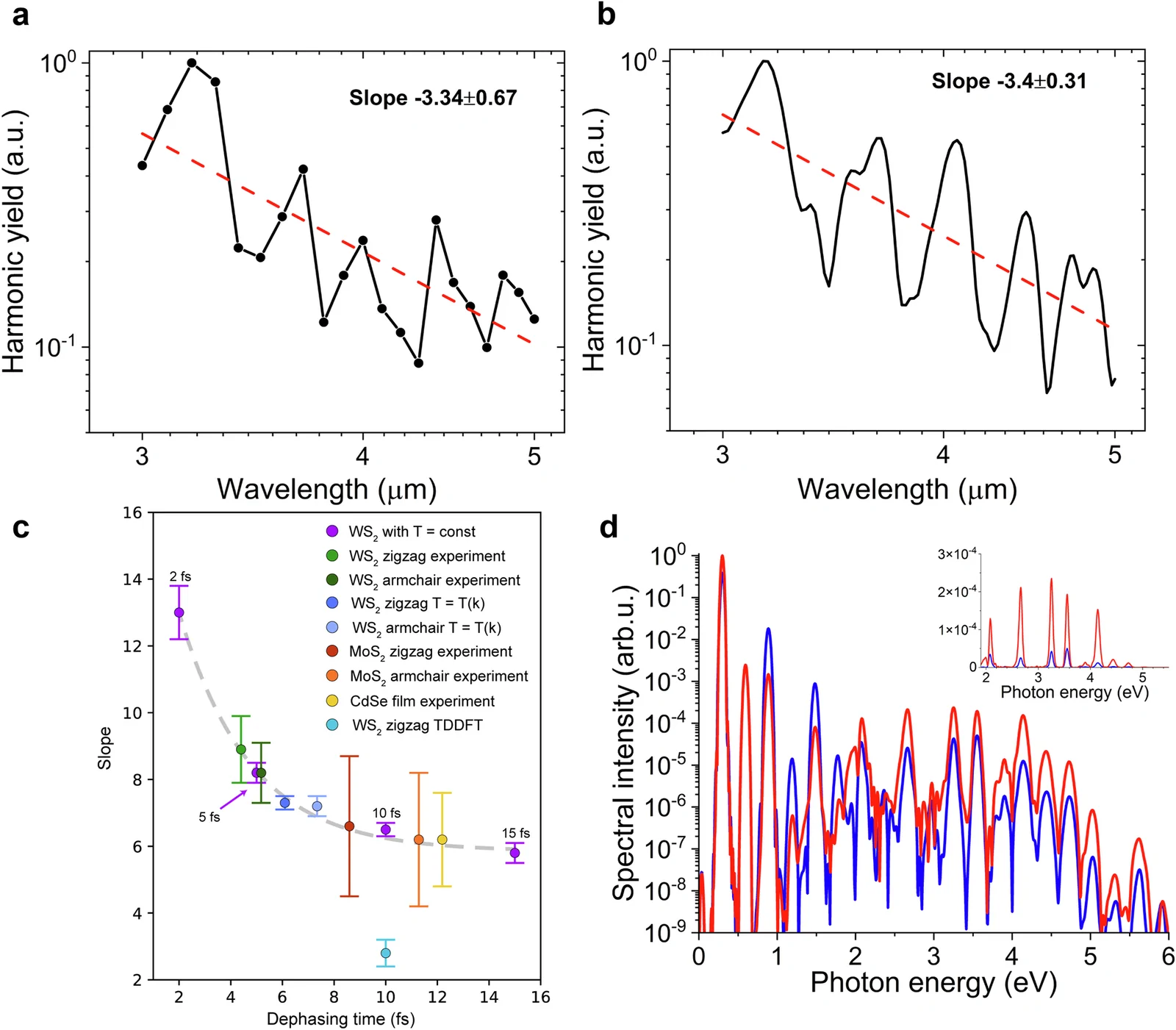
Summary of the numerical simulations with infinite and a finite constant dephasing. **a** rt-TDDFT and **b** 2D SBE numerical simulations without dephasing. The red line shows a fit with a power index given in the figure. Note the double log scale of the axes. **c** Summary of numerical calculations with constant dephasing time (magenta points). The dashed line shows the exponential fit. The slope values retrieved from the experimental measurements (green, dark green, red, yellow and orange points) and k-dependent simulations (blue and light blue points) are superimposed with constant dephasing time calculations for comparison. The error bars represent the error in fitting procedure. **d** Comparison of HHG spectra from 2D SBE numerical simulations with the best-to-experiment fit constant dephasing time *T*_2_ = 5 fs (blue curve) and ${T}_{2}({{\boldsymbol{k}}})$ (red line) for laser polarization oriented along armchair direction. The inset is for the spectral range measured in the experiments and plotted in linear scale.

To elucidate the impact of the dephasing time in HHG of solids, we conducted (i) ab-initio real-time time- dependent density functional (rt-TDDFT) calculations of HHG in a WS_2_ monolayer and (ii) numerically solved the two-dimensional semiconductor Bloch equations (SBE) with infinite dephasing time (see “Methods” and Supplementary Information Section 4 for details). The results of the simulations are shown in Fig. 3a, b. The results of SBE simulations are in excellent agreement with the results of rt-TDDFT calculations yielding a ${\lambda }^{-3.4}$ dependence (${\lambda }^{-3.3}$ dependence in rt-TDDFT calculations) and strong oscillations. However, both simulations did not include the dephasing time explicitly and failed to reproduce the much steeper experimentally measured dependence of the harmonic yield on the driving wavelength.

We also compared the experimental wavelength scaling to SBE simulations using a momentum-independent dephasing time, as commonly assumed in phenomenological descriptions of solid-state HHG. The cumulative results are shown in Fig. 3c. Consistent with previous work^34^, we find that the retrieved wavelength-scaling exponent depends strongly on the chosen constant value of ${T}_{2}$, and the dependence of the slope upon the value of the dephasing time can be well approximated (*R*-square is 0.9987) by the exponential function ${{{\rm{e}}}}^{-\tau /\triangle \tau }$ with the decrement $\triangle \tau \approx 2.76\pm 0.29$ (the dashed line in Fig. 3c). Thus, a suitably tuned constant ${T}_{2}$ can reproduce selected observables phenomenologically. However, it does not provide a microscopic explanation for why such ultrashort dephasing is required. Moreover, the value should be empirically adjusted not only for every material but also depending on the crystal orientation. By contrast, the momentum-dependent dephasing time ${T}_{2}\left(k\right)$ that we introduce later follows directly from the calculated scattering landscape and reproduces the observed scaling without invoking an ad hoc fitting parameter for each driving condition and material. This comparison shows that the main advance of the present work is not simply an improved fit, but a physically grounded interpretation of the effective HHG dephasing in terms of momentum-resolved scattering.

Finally, we compare the spectral shape and cutoff behavior in the harmonic spectrum for different laser wavelengths for ${T}_{2}$ = 5 fs (the constant dephasing value providing the best match for the wavelength dependence in the harmonic yield) and k-dependent ${{{\rm{T}}}}_{2}({{\bf{k}}})$. The exemplary spectra for 4.2 µm laser wavelength are shown in Fig. 3d. Briefly, we observe that the cutoff position remains roughly the same for both models of the dephasing. However, very short dephasing time values (roughly below 10 fs) result in strong suppression of the plateau structure at long driving wavelengths. In Fig. 3d, the cutoff harmonic's intensity is two orders of magnitude lower than the intensity of the 5th harmonic, the last harmonic with the energy of quanta below the bandgap. This strong plateau suppression at long wavelengths was not observed in our experiments; the 5th harmonic remains comparable or even weaker than the plateau harmonics. Also, the plateau suppression in numerical simulations with very short dephasing times was reported before and was argued as a strong indication that dephasing times below 10 fs result in the wrong structure of solid HHG spectra and propagation effects should be carefully taken into account instead^40^.

### Scattering processes and k-dependent dephasing time

To identify the physical origin of ultrafast dephasing, we recapitulate possible scattering processes occurring in solids: (i) The first collisional process, carrier multiplication, is much more efficient in 2D materials than in bulk semiconductors due to quantum confinement^41–43^. However, this process becomes efficient only if the excited electron has an excess energy equal or larger than the bandgap (*i.e*., a total energy larger than twice the bandgap), which is possible by single or several photon absorption from a light source with large photon energy. Under the conditions of strong-field excitation with photon energies much smaller than the bandgap, as it is realized in our experiments, the electron tunnels to the bottom of the conduction band, making carrier multiplication in TMDs inefficient and, thus, negligible. This was confirmed in experiments on transport phenomena under DC voltage where the carrier multiplication was identified primarily due to the carrier contribution from trap defects states^44,45^.

The second scattering process that can contribute to the dephasing in HHG is electron-electron scattering (ii). Electron-electron scattering depends sensitively on the number of excited carriers. Thus, if it were the dominant contribution for our experimental conditions, a substantial increase in the excited carrier density with laser intensity would be expected to modify the wavelength-scaling exponent significantly. Recent experiments on the influence of photodoping on HHG in 2D materials have shown that, under conditions of high density of the excited carriers, when screening effects can be neglected and electron-electron collisions play the major role, the dephasing time scales with the electron density as $\sim 1/{N}_{e}$^36^. Based on the Keldysh strong-field excitation rate in semiconductors^46,47^, we estimate that the carrier density increases by up to approximately one order of magnitude over the investigated intensity range. According to our numerical simulations and results from ref. ^34^, such a variation would be expected to induce a pronounced change in the effective dephasing time and hence in the scaling exponent. Experimentally, however, the extracted exponent changes by less than 10% when the laser intensity is varied by more than 70% (Fig. 2c). We therefore conclude that, under the excitation conditions studied here, carrier–carrier scattering is not the dominant contribution to the dephasing responsible for the steep wavelength scaling. This does not exclude a stronger role of carrier–carrier scattering at substantially higher excited-carrier densities, for example, in photo-doped^35–37^ or more strongly driven regimes.

**Fig. 4:**
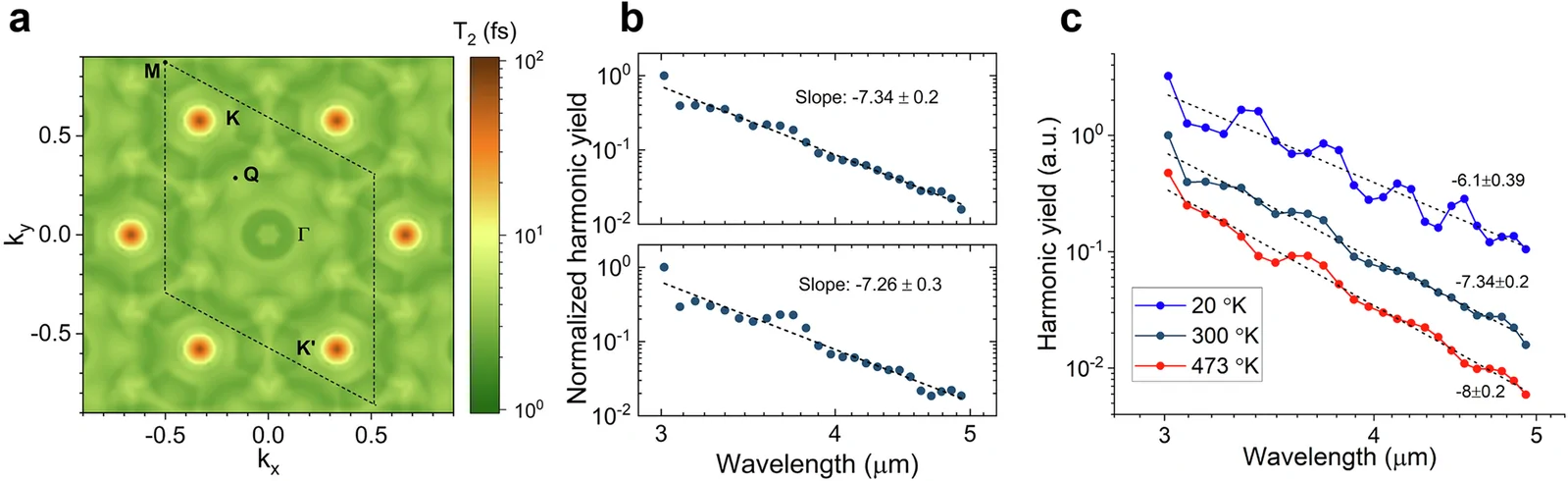
Numerical simulations for the k-dependent electron-phonon scattering time. **a** Two-dimensional map of the dephasing time between the first conduction band and the valence band, calculated for the 300 °K temperature. Note the logarithmic scale for the false colour, showing the scattering time. The short-dashed line marks the borders of the Brillouin zone. Also, high symmetry points are marked. **b** Normalised harmonic yield as a function of the pump wavelength for the zigzag (top figure) and the armchair orientations (bottom figure), calculated by SBE with T_2_ = T_2_(**k**) shown in **a**. **c** Wavelength dependence of the harmonic yield for different lattice temperatures. The dotted lines are the linear fits with the slopes marked in the figure.

Finally, we consider the contribution of electron-phonon scattering (e-ph scattering, iii). Using DFT methods, we calculate the total (summed over all phonon modes) band-specific electron-phonon scattering time in WS_2_ (see Supplementary Information Section 3 for details). From the band-specific scattering times, we introduce the ***k***-dependent polarization dephasing time ${T}_{2,{mn}}\left({{\boldsymbol{k}}}\right)$ between two bands m and n as $\frac{1}{{T}_{2,{mn}}({{\boldsymbol{k}}})}=\frac{1}{2}\left(\frac{1}{{{{\rm{\tau }}}}_{m}({{\boldsymbol{k}}})}+\frac{1}{{{{\rm{\tau }}}}_{n}({{\boldsymbol{k}}})}\right)$. The corresponding dephasing time ${T}_{2}\left({{\boldsymbol{k}}}\right)$ between the valence and the first conduction bands is shown in Fig. 4a. The striking feature of the dephasing time is its pronounced dependence on the electron lattice momentum. At the excitation point in the K-valley (or K′-valley), it is about 70 fs and drops abruptly to below 10 fs as soon as the electron leaves the valley and reduces to ≈4 fs in the vicinity of the Γ-point (Fig. 4a). This dephasing time enters the two-dimensional SBE (see “Methods” and Supplementary Information Section 4 for details) to simulate HHG. Figure 4b displays such calculated wavelength dependence of the harmonic yield for the laser pulse parameters and the spectral integration range used in the experiments. The retrieved slope of 7.3 ± 0.2 (7.2 ± 0.4) for the zigzag (armchair) orientation agrees rather well with the values retrieved from the experimental measurements. The remaining mismatch between experimental and simulated slope values likely reflects several factors. First, as shown previously^34^ and confirmed by our simulations (Fig. 3c), the wavelength scaling exponent is highly sensitive to the precise magnitude of the dephasing time. Second, the electron–phonon scattering rates used here are evaluated within a field-free perturbative framework. Under strong driving, dynamically dressed bands, nonthermal carrier distributions, and additional many-body channels may renormalize the effective scattering and dephasing rates. Noteworthy, the simulations based on the k-dependent scattering rates at higher lattice temperature (473 °K in Fig. 4c) essentially reproduce the experimentally determined slope value, suggesting that indeed nonthermal or some effective hot carrier distribution can change the scattering rates significantly. The present treatment should therefore be viewed as a first-principles-informed effective description for the experimentally studied regime. Its success in reproducing the measured trends nevertheless indicates that momentum-resolved electron–phonon scattering captures the leading contribution to ultrafast dephasing under these conditions.

The numerical simulations also reproduce the oscillations in the dependence of the harmonic yield on the driving wavelength (Fig. 4b,c). These oscillations were attributed before to quantum path interference for HHG in gases^48^ and solids^34^. Detailed investigation of these oscillations is the subject of separate work. Here, we would like to note that the amplitude of the oscillations is directly linked to the value of the dephasing time: Larger values of the dephasing time result in increased amplitudes of the oscillations. Therefore, the modulation amplitude might be used as well as an indicator of the dephasing process’ timescales.

The identified origin of the dephasing as e-ph scattering suggests that the dephasing time might be temperature-dependent. Indeed, numerical simulations performed for a lattice temperature of 20 ˚K predict that the scattering time increases in the K (K’) valley up to 1 ps and by a factor 2-3 across the band, remaining ≈3 fs near the Γ- point (see Supplementary Fig. 6). The calculated scattering time at low temperatures suggests that the processes of phonon emission in electron-lattice collisions play the dominant role. Also, the increased values at low temperatures predict a significant increase in the harmonic yield. This is indeed confirmed by the numerical simulations, showing a reduction in the power index and up to one order of magnitude increase of the harmonic yield at long wavelengths (Fig. 4c). Noteworthy, also the amplitude of the oscillations increases significantly, mirroring longer dephasing times. Thus, our simulations suggest a possibility for a direct experimental determination of the contributions of the electron-electron and electron-phonon scatterings by conducting wavelength- and temperature-dependent HHG measurements for different laser intensities. Remarkably, a very recent publication on temperature-dependent measurements of HHG in crystalline silicon confirms our predictions, demonstrating a strong increase in harmonic yield at low temperatures for a fixed driving wavelength and explaining it by electron-phonon contribution to the dephasing processes^49^.

**Fig. 5:**
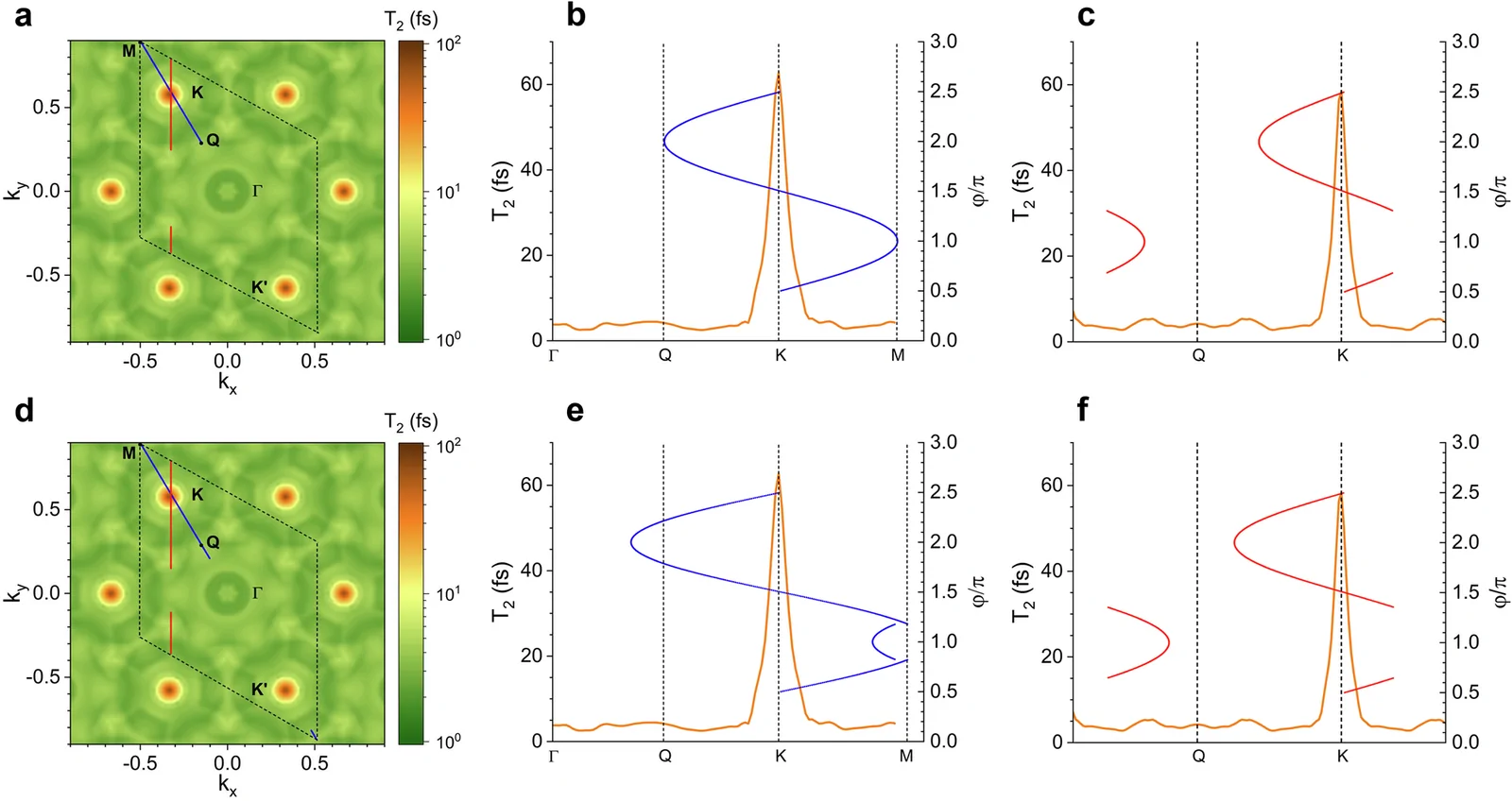
Trajectory resolved dephasing. **a** Electron trajectory in k-space for the laser wavelength 3.5 µm and the polarization oriented along the zigzag symmetry direction (blue line) and armchair direction (red line). **b** The 2D profile of the dephasing time along the zigzag direction (orange curve) and the corresponding electron trajectory (blue line). **c** The 2D profile of the dephasing time along the armchair direction (orange curve) and the corresponding electron trajectory (red line). **d**–**f** The same as **a**–**c** for 4.5 µm laser wavelength.

The calculated scattering times reveal a strong dichotomy between near-edge and strongly displaced states in momentum space. Near the band extrema, the scattering time remains on the order of 100 fs, whereas carriers driven away from these regions encounter scattering times below 10 fs and, in parts of the Brillouin zone, only a few femtoseconds. The HHG-relevant coherence time is therefore not only a single material constant but an effective trajectory-dependent average over the regions of momentum space sampled during the strong-field motion. In this sense, the few-femtosecond dephasing commonly required in HHG simulations emerges naturally from the underlying momentum-resolved scattering landscape. To demonstrate this, we performed a trajectory- resolved analysis of the dephasing time for a monochromatic driving laser field. There, the electron trajectory in the conduction band is ${{\boldsymbol{k}}}\left(\varphi \right)={{{\boldsymbol{k}}}}_{0}+\frac{e{{{\boldsymbol{E}}}}_{0}}{\hslash \omega }\left(\cos \varphi -\cos {\varphi }_{0}\right)$ where ${{{\boldsymbol{k}}}}_{0}$ is the initial lattice momentum of the electron at the K-point, ${{{\boldsymbol{E}}}}_{0}$ and ω are the electric field strength vector and the angular frequency in the monochromatic laser wave, φ and ${\varphi }_{0}$ are the laser field phase and the phase of the electron birth correspondingly. Note that (a) lattice momentum conservation requires the hole to be excited in the K′ point and (b) for linear polarization, the electron is excited with equal probability also at the K′ point (the corresponding hole at the K-point). In Fig. 5 the exemplary electron trajectory, starting at the K point at the field phase (“birth” phase) ${\varphi }_{0}=\pi /2$ for a driving wavelength of 3.5 µm (Fig. 5a) and 4.5 µm (Fig. 5d), is superimposed with the dephasing time landscape. Using the dephasing time profile along the selected electron trajectory, we can calculate the cycle average, trajectory-specific dephasing time as $\langle {T}_{2}\rangle ({\varphi }_{0})=\frac{2\pi }{{\int }_{0}^{2\pi }{d}_{\varphi }{T}_{2}({{\boldsymbol{k}}}(\varphi,{\varphi }_{0}))}$. For the exemplary trajectories in Fig. 5, we calculate $\left\langle {T}_{2}\right\rangle$ values ≈ 4 fs for both, 3.5 µm and 4.5 µm driving wavelengths for the motion along zigzag direction and 4.2 fs and 4.4 fs for the motion along the armchair direction, correspondingly. Thus, the HHG-relevant effective coherence time is in the few-fs regime, despite much longer near-edge scattering times.

Finally, simulations for monolayer MoS_2_ provide very similar results with slightly larger scattering times, which may explain the difference in the slopes retrieved for MoS_2_ and WS_2_ monolayers. Also, for CdSe in wurtzite structure, as for the TMD monolayer, the scattering time is strongly k-dependent and drops abruptly from ≈100 fs at the Γ-point to a few femtoseconds for larger lattice momenta (see Supplementary Fig. 7). Therefore, the ultrashort nature of the e-ph scattering outside the bandgap minima is a universal phenomenon in solids, now experimentally assessable by the nonlocal processes of HHG.

Concluding this section, we emphasize that the wavelength scaling of the HHG yield is not determined by dephasing alone. It also reflects the band structure, the driven trajectory length in momentum space, and the relative interband and intraband contributions. However, within our multi-band full-Brillouin zone simulations, the steepness of the experimentally observed scaling is controlled primarily by the dephasing term: calculations without dephasing or with long constant dephasing time yield substantially weaker scaling, whereas introducing momentum-resolved dephasing reproduces the observed trend. We therefore interpret dephasing as the dominant contribution governing the scaling exponent in the present experimental conditions, while other ingredients shape the detailed spectral response.

## Discussion

Summarizing, we found experimentally that the high-harmonic yield in two-dimensional and bulk semiconductors sharply decays with increasing laser wavelength, revealing material-specific scaling exponents (approximately ${\lambda }^{-6}$ to ${\lambda }^{-9}$) steeper than those in atomic gases^38^. Our combined experiments and simulations show that the steep wavelength dependence of the HHG yield in the investigated semiconductors is consistent with ultrafast, momentum-dependent dephasing during strong-field electron–hole dynamics. The calculations indicate that although scattering near the band extrema is relatively weak, laser-driven carriers are rapidly pushed into regions of the Brillouin zone where electron–phonon scattering becomes much stronger, resulting in an effective HHG dephasing on the few-femtosecond timescale. In the materials and excitation regime studied here, this provides a microscopic explanation for the ultrashort dephasing commonly introduced phenomenologically in SBE models. By incorporating a momentum-dependent dephasing time ${T}_{2}({{\boldsymbol{k}}})$ derived from first-principles scattering rates, we connect the phenomenological dephasing used in previous HHG simulations to an underlying momentum-resolved scattering landscape. This offers a physically grounded framework for interpreting why strong-field HHG appears to require an order of magnitude shorter coherence times than weak-field optical measurements near the band edge. At the same time, the effective dephasing remains condition-dependent: it is expected to vary with the portion of the Brillouin zone sampled by the driven carriers, and may also depend on temperature, carrier density and driving strength. More broadly, our results suggest that momentum-resolved scattering should be considered when modeling strong-field coherence in solids. Whether electron–phonon scattering remains dominant in other materials or under substantially higher carrier densities will depend on the experimental regime; in particular, carrier–carrier scattering may become increasingly important at stronger excitation or under deliberate photodoping.

Looking forward, these results have significant implications for both fundamental and applied photonics. First, knowing that electron–phonon scattering sets the fundamental limit to coherence in solid HHG enables new strategies to mitigate decoherence. For instance, material engineering or environmental control (such as cryogenic operation to reduce phonon activity) could extend the effective ${T}_{2}$ and boost harmonic efficiency. Such control over ultrafast dephasing would directly promote the development of compact attosecond pulse sources and frequency combs in solids, whose performance is currently constrained by limited harmonic yield. Second, and crucially, suppressing electron–phonon dephasing opens a pathway to preserve quantum coherence in the HHG process. This could allow the generation of nonclassical light at high photon energies and fluxes, effectively turning solid-state HHG into a bright source of entangled photons and squeezed light. By leveraging materials with weaker electron–phonon coupling or dynamically altering phonon interactions (e.g., via phononic excitation or strain), one could envision solid-state high-harmonic devices that produce large numbers of entangled photons on ultrafast timescales – a powerful resource for quantum optics and communication. In summary, by uncovering and controlling the electron–phonon decoherence in strong-field-driven solids, our work paves the way toward scalable quantum photonic technologies based on high-harmonic generation. The convergence of strong-field physics with quantum optics promises not only deeper insight into light–matter interaction at extreme conditions but also practical advances in ultrafast quantum light sources built on solid-state platforms.

## Methods

### Experiments

A Ti:sapphire-based regenerative amplifier (Coherent Astrella, 800 nm central wavelength, 35 fs pulse duration at 1 kHz repetition rate) was used to pump an optical parametric amplifier (Light Conversion TOPAS), followed by a noncollinear difference frequency generation (NDFG) module, offering ultrashort pulses with the duration from 60 to 100 fs in a spectral region from 3 to 12 µm. To ensure that the wavelength-dependent measurements were conducted under fixed peak intensity conditions, the pulses were characterized temporally, using the second harmonic frequency-resolved optical gating (SH-FROG) method, and spatially, using a mid-IR CCD camera for measuring a focal intensity distribution. The harmonics were measured in the 200–1100 nm wavelength range, limited by the spectrometer and the detector. All spectra were corrected for the wavelength-dependent response of the spectrometer and detector, converted to the frequency domain, and integrated over the spectral region above the material bandgap within the experimentally accessible window. The resulting integrated quantity is referred to throughout the manuscript as the HHG yield. The same spectral windows were used when comparing experimental and simulated yields. To keep the fixed intensity value firmly, the dependence of the yield on the pump energy was measured for all wavelengths. The resulting yield for each laser wavelength was interpolated as a function of the laser peak intensity, and the final data were acquired from the interpolated data (see Supplementary Information Section 2). The experimental setup is shown in Supplementary Fig. 2. A laser beam was attenuated by a pair of wire-grid polarisers on a silicon substrate and focused by a 50 mm CaF_2_ lens at a normal incidence onto a TMD/CdSe sample deposited on a c-cut sapphire substrate. Pump polarization was tuned by a broadband half-wave plate (B-Halle). Harmonic emission is collected by a CaF_2_ *f* = 25 mm lens in transmission geometry and measured by a Kymera-328i spectrometer equipped with a cooled UV-enhanced CCD camera. An on-site microscopy setup with an x50 objective lens (NUV Plan Apo 0.45 NA) was implemented for optical diagnostics and alignment of the samples. All experiments were performed under ambient conditions.

### Numerical simulations

The geometry and the electronic structure of single-layer WS_2_ were calculated using density functional theory as implemented in Quantum Espresso^50^. Phonon properties were obtained within density functional perturbation theory using the same software. The optimized lattice constant was found to be $a=3.188{{{\AA }}}$ in agreement with previously reported results^51,52^. The calculated bandgap is ${E_{g}}=1.86 \, {\rm{eV}}$ and therefore smaller than the experimentally observed value ($\approx 2 \, {\rm{eV}}$). For all calculations, we used the SG15 optimized norm-conserving pseudo-potentials^53,54^ from http://www.quantum-simulation.org/potentials/sg15_oncv/index.htm. A kinetic energy cutoff for the wavefunction of 90 Ry was applied, and the PBE functional was used in all cases^55^. To ensure a sufficient decoupling of adjacent layers, we added about 19.4 Å of vacuum in the direction perpendicular to the layer. For the electron and phonon calculations, we used Monkhorst–Pack meshes with 18 × 18 × 1 and 9 × 9 × 1 points, respectively. The electronic band-structure and the phonon dispersions are shown in Supplementary Fig. 4. The electron-phonon induced finite lifetime of the electronic states was obtained using the electron-phonon Wannier functions (EPW) approach^56,57^. It is calculated from the imaginary part of the electron-phonon self-energy for the respective Bloch state as in ref.^58^. For the integration over the Brillouin zone, we used 27 × 27 × 1 *k*-points and a very fine mesh of 500 × 500 × 1 *q*-points to assure convergence.

The rt-TDDFT calculations were carried out using the Octopus code^59^ with the “hgh_lda” pseudopotentials, the semicore set of Hartwigsen–Goedecker–Hutter in the local density approximation^60^. The simulations converged for a Brillouin zone sampling of 40 × 40 and a real-space mesh with 0.3 Bohr spacing.

For the SBE simulations, we followed the approach outlined in ref.^2^. To calculate the energy bands and transition dipole elements as well as Berry connections, we used a three-band tight-binding model^61^ including one valence and two conduction bands and spin-orbit coupling. To incorporate scattering into the SBE model, we apply the calculated band- and momentum-resolved electron–phonon scattering rates to the interband polarization dynamics. Specifically, we treat scattering as a source of polarization dephasing rather than explicitly as a redistribution of carrier occupations. This approximation is motivated by the fact that the HHG yield in the present regime is more sensitive to the coherence of the interband polarization than to the detailed nonequilibrium redistribution of populations. Within a Markovian effective description of scattering-induced decoherence, the dephasing rate of the polarization between bands (m) and (n) is constructed from the corresponding microscopic scattering rates, yielding a momentum-dependent effective dephasing time ${T}_{2}({{\boldsymbol{k}}})$. This mapping does not include all possible contributions to decoherence, such as pure dephasing or other many-body effects beyond the present treatment and should therefore be interpreted as an effective physically motivated correspondence rather than an exact identity. The details of SBE model are elaborated in the Supplementary Information Section 4.

## Supplementary information

Supplementary Information

Description of Additional Supplementary Files

Dataset 1

Transparent Peer Review file

## Data Availability

The numerical data underlying the wavelength-dependent integrated harmonic-yield measurements presented in Fig. 2 are provided with this paper as Supplementary Data 1.

## Code availability

The first-principles calculations were performed using the publicly available Quantum ESPRESSO⁵⁰, EPW⁵⁶˒⁵⁷ and Octopus⁵⁹ software packages. The custom codes used for numerical simulations, data processing and analysis are available from the corresponding author upon request.
