# Supplementary material for: Strong field spectroscopy of many-body interactions in solids
Source: Nat Commun. 2026 Oct 20;17:10954. doi: 10.1038/s41467-026-78716-1 (PMC13680610; doi:10.1038/s41467-026-78716-1)
Supplement: Supplementary file 1 [file 41467_2026_78716_MOESM1_ESM.pdf]

Supplementary Information for
Strong Field Spectroscopy of Many-Body Interactions in Solids

Viacheslav Korolev, Thomas Lettau, Vipin Krishna, Alexander Croy, Martin Thümmler, Michael Zürch, Christian Spielmann, Maria Wächtler, Ulf Peschel, Stefanie Gräfe, Giancarlo Soavi and Daniil Kartashov

S1. Sample Fabrication and Characterisation

MoS_2 and WS_2 monolayer samples were prepared by mechanical exfoliation from a commercial bulk synthetic crystal (HQGraphene) using an adhesive tape (Bluetape Minitron) and subsequently transferred to sapphire substrates by the PDMS (Gel-Pak) dry transfer method [1]. The samples were identified by optical contrast, and their monolayer nature was confirmed by micro-photoluminescence (PL) spectroscopy in reflection geometry from a SiO_2/Si substrate. PL measurements were performed with CW excitation (Cobolt 08-DPL 532nm) using a 50x objective with a numerical aperture of 0.42 (Mitutoyo Plan Apo) resulting in a focal spot size of ~1μm. We observed PL maxima at ~ 616nm (~2.01 eV) / 629nm (~1.97 eV) corresponding to the A-exciton/trion of WS_2 (Supplementary Fig.1a), and at ~ 652nm (~1.9 eV) / 602nm (~2.05 eV) / 665nm (~1.86 eV) corresponding respectively to the A-exciton, B- exciton and trion of MoS_2 (Supplementary Fig. 1), in agreement with literature [2,3].

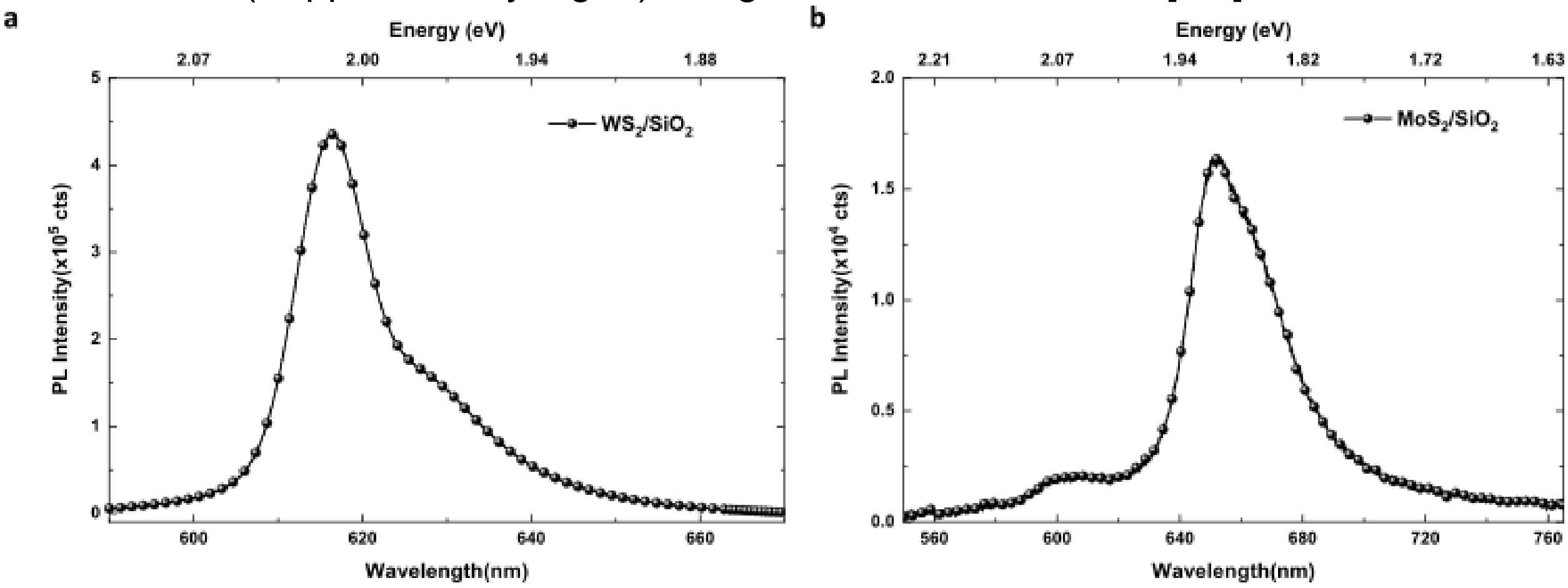

Supplementary Fig. 1. Photoluminescence characterisation of monolayer (a) WS_2 and (b) MoS_2 on SiO_2/Si substrates.

S2. Experimental setup and methods

S2.1. Experimental setup

The experimental layout is shown schematically in Supplementary Fig. 2. A commercial femtosecond Ti:Sapphire laser system, operating at 0.8 μm wavelength at 1 kHz repetition rate and delivering 35 fs laser pulses with the energy up to 4.6 mJ (Coherent Astrella), was used to pump an optical parametric amplifier (OPA) tuneable in the range 1150-1600 nm and 1600-2600 nm for the signal and idler waves correspondingly (TOPAS, Light Conversion). The difference frequency generation module (NDFG, Light Conversion) delivers the mid-IR laser pulses, tuneable in the spectral range 3-12 μm with pulse duration 60-100 fs. The polarization of the mid-IR pulses and the pulse energy were controlled by a pair of wire-grid polarizers. A removable mirror was used to intercept the mid-IR beam for focal spot and pulse characterization.

To characterise the focal intensity, we used a long-focusing CaF_2 lens (200 mm) on a translation stage and measured with a MID-IR CCD camera (Data Ray) the spot size as a function of the lens position. The spatial integration of the recorded focal spot image was used then to determine the peak intensity in the beam. Assuming negligible chromatic aberrations, the retrieved focal intensity was recalculated for the 50 mm

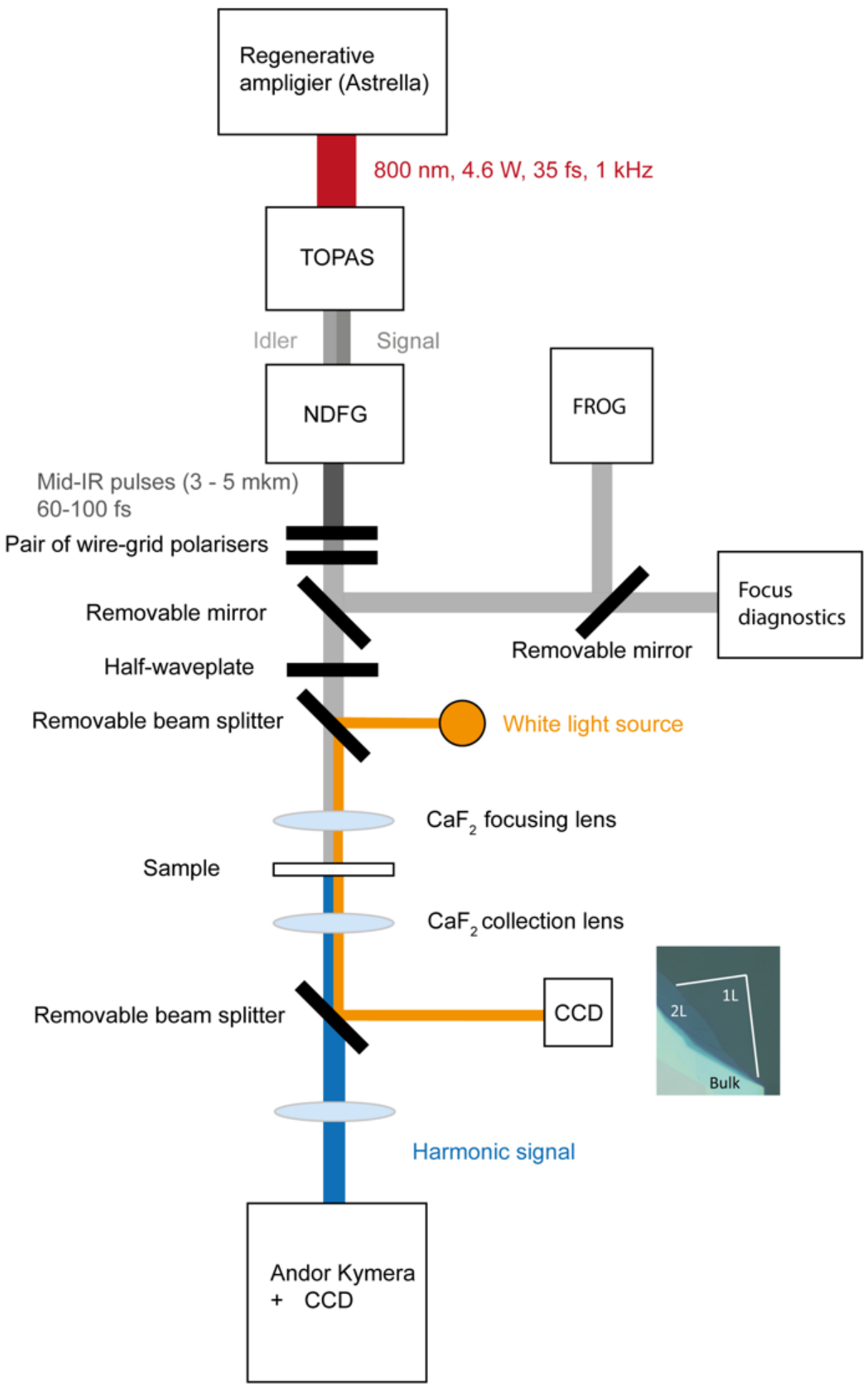

Supplementary Fig. 2. Schematic presentation of the experimental setup.

CaF_2 lens used to focus the beam on the sample.

The temporal characterization of the pulses was carried out using the second harmonic generation frequency-resolved optical gating technique (SHG FROG). A CaF_2 optical window of the corresponding thickness was placed at the FROG entrance to mimic the focusing lens dispersion. In general, the pulse dispersion management was carried out by inserting optical windows of different thickness and materials (Si, CaF_2, YAG) in the beam and minimizing the retrieved pulse duration. Additionally, the dispersion was optimized based on the high-order harmonic spectral shape and intensities. Removable beamsplitters together with a white light source and removable 50x objective were used for on-site microscopy of the sample. The harmonic spectra were measured by a Kymera-328i spectrometer equipped with a cooled UV-enhanced CCD camera.

S2.2. Fixing the Laser Intensity for all Wavelengths

To analyse the wavelength dependence, it is essential to keep the peak intensity of the laser pulse fixed. To achieve this, we first performed harmonic-yield measurements as a function of an average power together with FROG traces and focal intensity distributions (Supplementary Fig. 3a and c) for every wavelength in the experiment. When the pulse is characterised in space and time domain, we can retrieve the harmonic yield as a function of the peak intensity. This dependence is a smooth

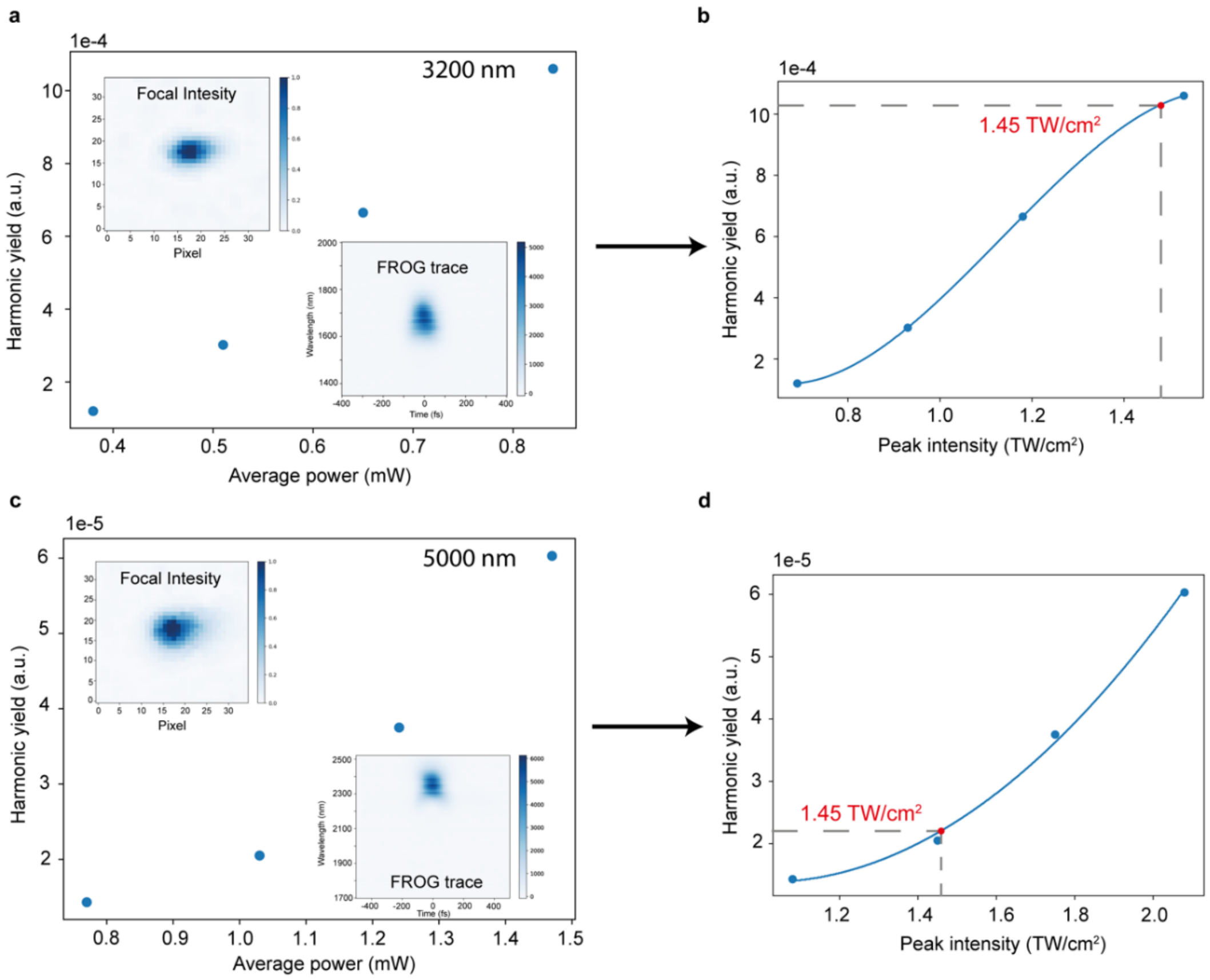

Supplementary Fig. 3. Calibration of laser intensity. **a** Harmonic yield as a function of a laser average power, measured in the experiment, inset figures represent a 2D focal intensity distribution and FROG trace at 3200 nm central wavelength. **b** Harmonic yield as a function of peak laser intensity, retrieved according to FROG traces and focal distribution. The solid line shows a polynomial fit, and the dashed grey line represents the choice of a fixed intensity. **c** and **d** Same but for 5000 nm central wavelength.

function and can be fitted by a polynomic function (Supplementary Fig. 3b and d). Thus, choosing the intensity for one of the laser wavelengths, we interpolated the harmonic yield for all other wavelengths, using the retrieved polynomial fits, as it is shown in Supplementary Fig. 3.

S3. Electron-Phonon Scattering Rates

Supplementary Fig. 4a shows the calculated electronic band-structure $\varepsilon_n(\boldsymbol{k})$ for a selection of bands around the Fermi energy. The phonon dispersion $\omega_\lambda(\boldsymbol{q})$ in Supplementary Fig. 4b displays the quadratic dispersion of the flexural mode which is characteristic for 2D materials [4].

To verify the quality of the Wannierisation procedure which is needed for the computation of the electron-phonon self-energy, we compare the band structure and the phonon dispersions obtained with electron-phonon Wannier functions (EPW) [5,6] to the respective results from Quantum Espresso (QE) [7] simulations. As follows from Supplementary Fig. 4, there is a very good agreement between the original (QE) and the interpolated data (EPW). The electron-phonon scattering rate

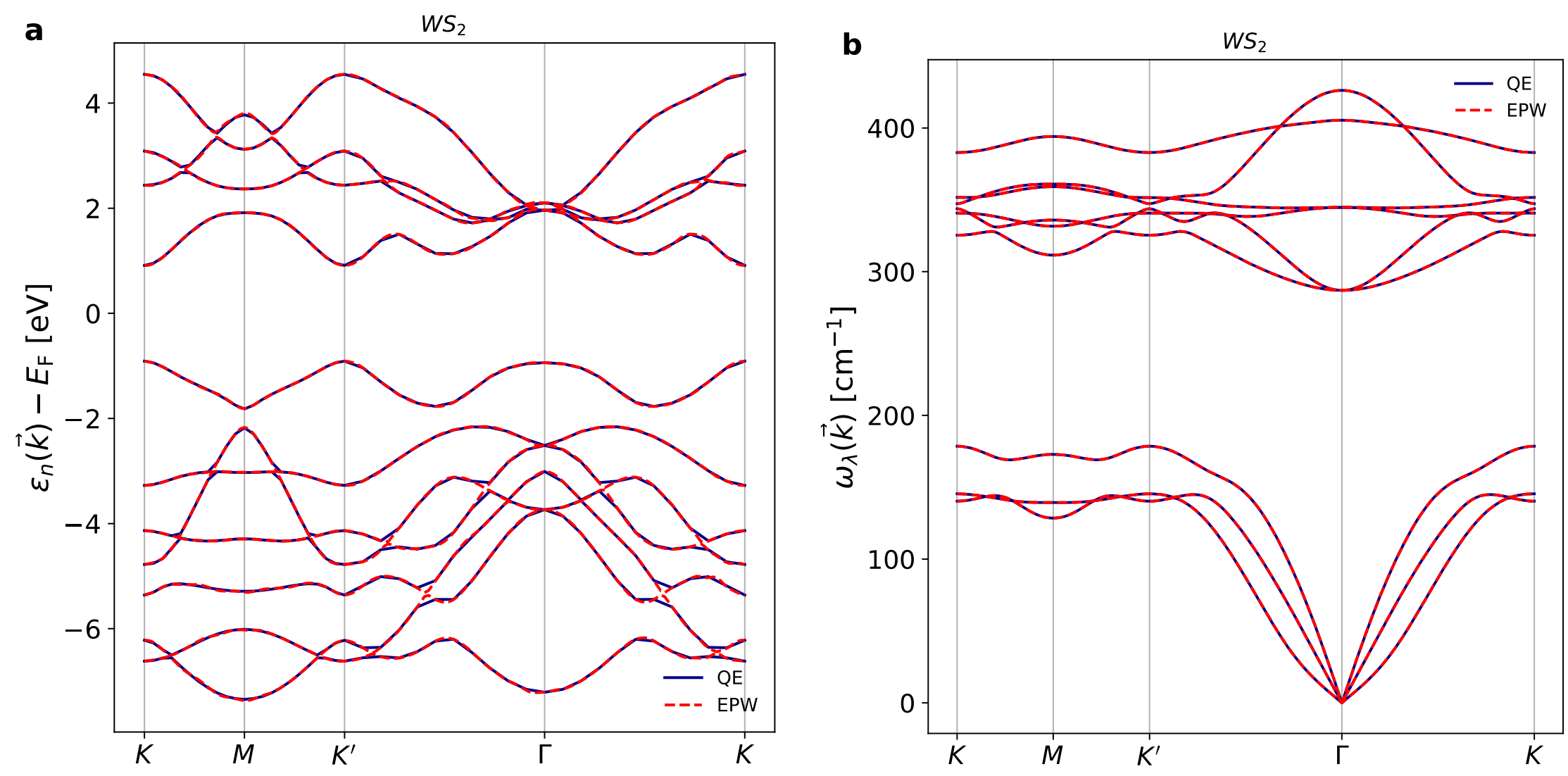

Supplementary Fig. 4. Comparison of **a**) the electronic band-structure and **b**) the phonon dispersions calculated with Quantum espresso (QE, blue solid lines) and EPW (red dashed lines).

$1/\tau_n(\boldsymbol{k})$ is obtained from the first order electron-phonon self-energy as follows [8]:

$$\frac{1}{\tau_n(\boldsymbol{k})} = \frac{2\pi}{\hbar} \sum_{n,\vec{q},\lambda} \left|g_{nm}^{\lambda}(\boldsymbol{k},\boldsymbol{q})\right|^2 \{\left[n_\lambda^0(\boldsymbol{q}) + f_n^0(\boldsymbol{k}+\boldsymbol{q})\right]\delta(\varepsilon_n(\boldsymbol{k}+\boldsymbol{q}) - \hbar\omega_\lambda(\boldsymbol{q}) - \varepsilon_m(\boldsymbol{k})) + \left[n_\lambda^0(\boldsymbol{q}) + 1 - f_n^0(\boldsymbol{k}+\boldsymbol{q})\right]\delta(\varepsilon_n(\boldsymbol{k}+\boldsymbol{q}) + \hbar\omega_\lambda(\boldsymbol{q}) - \varepsilon_m(\boldsymbol{k}))\}.$$

Here, $g_{nm}^{\lambda}(\boldsymbol{k},\boldsymbol{q})$ is the electron-phonon scattering matrix element, $f_n^0(\boldsymbol{k})$ and $n_\lambda^0(\boldsymbol{q})$ denote the Fermi and Bose distributions for electrons and phonons, respectively. Thus, the total scattering rate is a sum over the scattering rates for each phonon mode, populated according to the Boltzmann distribution at a given temperature, and including both, phonon absorption and phonon emission processes. The electron-phonon scattering time $\tau_n(\boldsymbol{k})$ for the first conduction and the highest valence bands is shown in Supplementary Fig. 5 for the ambient temperature of 300 °K. The temperature dependence of the scattering time is shown in Supplementary Fig. 6 where the lineouts along the main symmetry directions for the valence and the conduction bands are shown for the ambient temperature of 300 °K and cryogenic temperature of 20 °K. To demonstrate the ultrafast nature of the electron-phonon scattering in solids, Supplementary Fig. 7 shows the scattering rate along main symmetry directions in a wurtzite CdSe crystal.

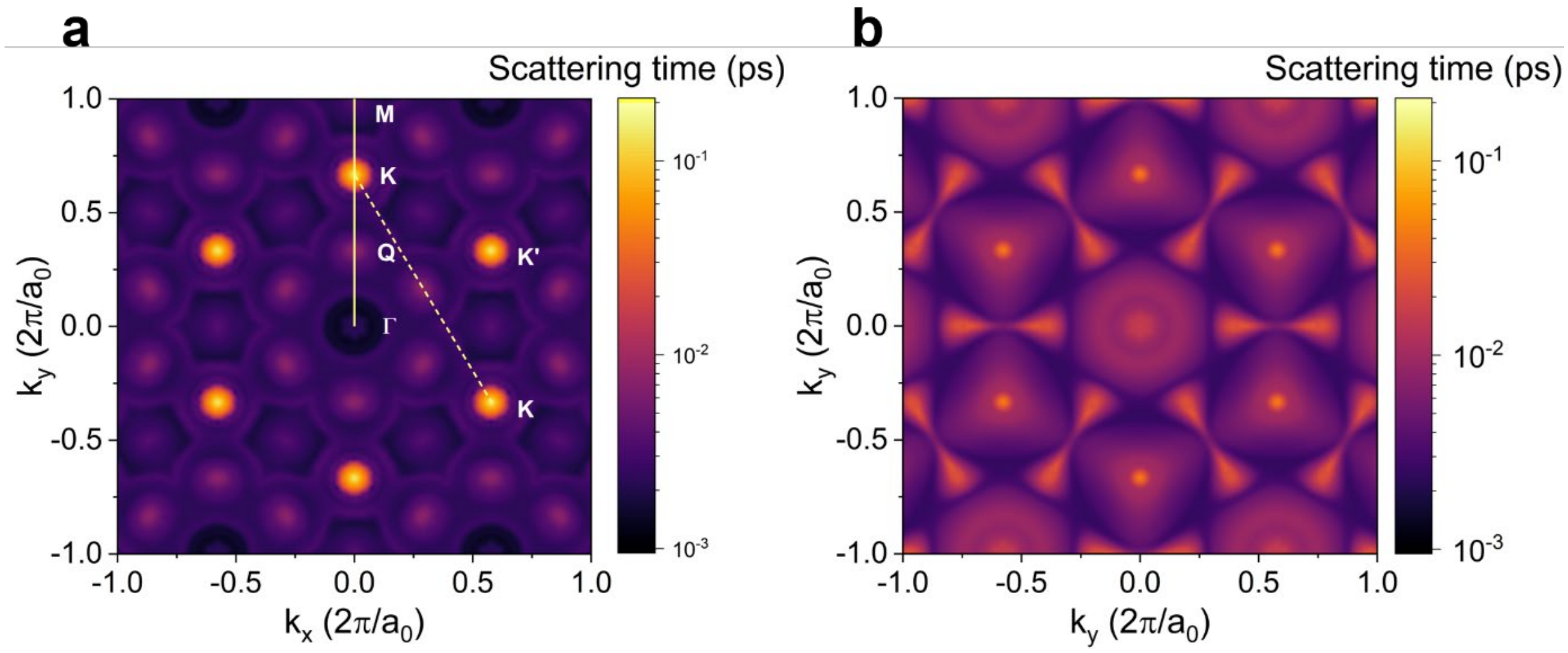

Supplementary Fig. 5. Two-dimensional map of **a** electron-phonon scattering time in the first conduction band and **b** hole-phonon scattering time in the valence band. Note the logarithmic scale for the false colour, showing the scattering time. The solid line depicts electron motion in the band along zigzag direction, the dotted line – along the armchair direction. Also, high symmetry points are marked.

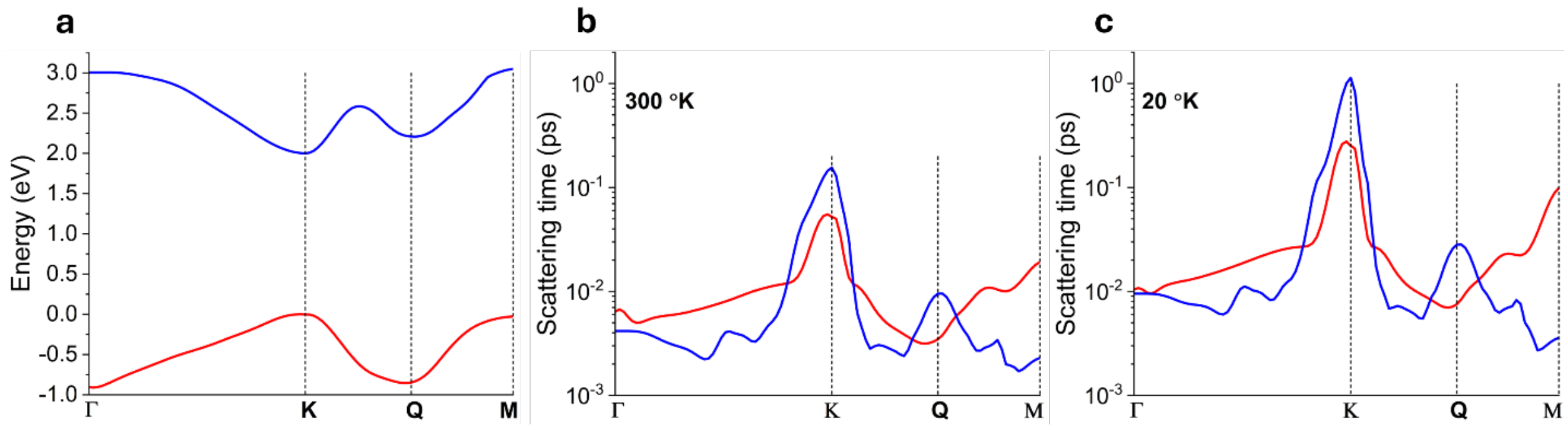

Supplementary Fig. 6. Temperature dependence of the electron-phonon scattering. **a** WS_2 band structure along main symmetry directions. The red line is the valence band, the blue line is the first conduction band. **b** k-dependent electron-phonon scattering time for the upper valence band (red line) and the first conduction band (blue line) calculated at the 300 °K. **c** k-dependent electron-phonon scattering time for the upper valence band (red line) and the first conduction band (blue line) calculated at the 20 °K. Note the logarithmic scale for the plots.

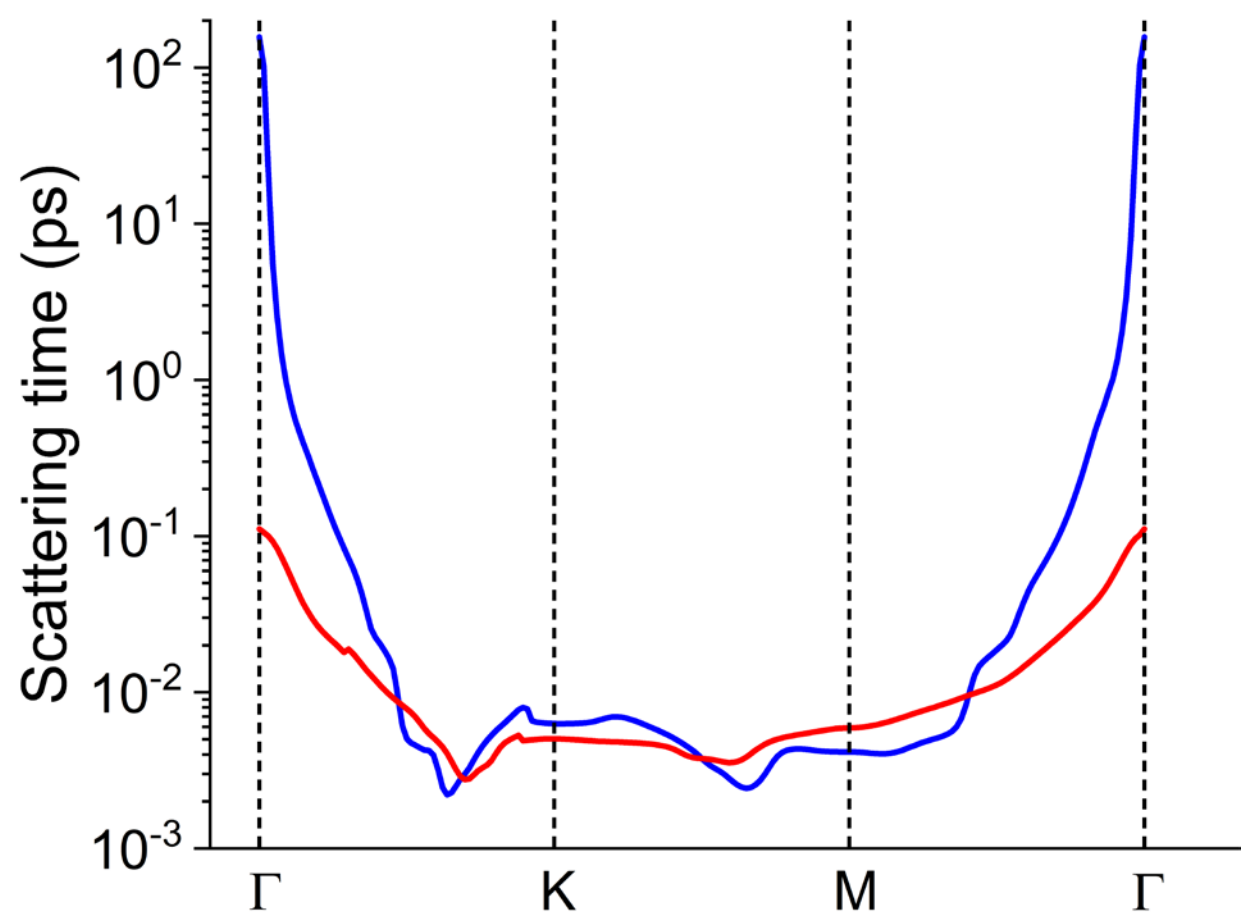

Supplementary Fig. 7. k-dependent electron-phonon scattering time for the upper valence band (red line) and the lowest conduction band (blue line) in wurtzite CdSe crystal at 293 K temperature.

S4. HHG Simulations with Semiconductor Bloch Equations

A concise overview of different approaches using SBE can be found in [9]. Here we only refer to changes and modifications introduced to the standard approach. In our simulations of HHG with the SBE approach, we consider the upper valence and the two lowest conduction bands. The choice of these three bands is justified by the spectral range of harmonics measured in the experiments. When including the k-dependent electron-phonon scattering processes in the SBE equations, we consider the effect of the scattering only on the polarisation, and not on the occupation number. The full treatment of carrier scattering would require a kinetic approach based on two-dimensional the Boltzmann equations, which is computationally extremely demanding [10]. However, the redistribution of occupation numbers plays a minor role in the HHG yield compared to the polarisation coherence, and thus, such a treatment is not needed. Therefore, we treat scattering in the context of the Lindblad formalism where each scattering event with a rate $\gamma = \tau^{-1}$ is known to create a dephasing of the associated polarisation proportional to $\gamma/2$ (see e.g. [11]).

This can be understood as follows. We start from the Lindblad equation [12]

$$\partial_t \rho = -i[H_0, \rho] + \sum_i \gamma_i \left(L_i \rho L_i^\dagger - \frac{1}{2} L_i^\dagger L_i \rho - \frac{1}{2} \rho L_i^\dagger L_i \right),$$

where the first term is the normal von-Neumann equation for the density matrix describing the system, the γ_i are the scattering rates and the L_i are the so-called collapse operators, which describe the dissipative processes. Since we are not interested in the actual scattering but only in its effect on the microscopic polarization, we assume that we can describe the collapse operator and scattering rates by

$$L_i \to L_{n\mathbf{k}} = |s\rangle\langle\phi_{n\mathbf{k}}|, \;\; \gamma_i \to \frac{1}{\tau_{n\mathbf{k}}},$$

where $|\phi_{n\mathrm{k}}\rangle$ is the Bloch state for band n at momentum $\mathbf{k}$ and $|s\rangle$ a fictious empty state in which all scattered electrons will disappear. Applying the sum over the collapse operators to a microscopic polarization, e.g., to $|\phi_{m\mathbf{k}}\rangle\langle\phi_{n\mathbf{k}}|$ yields

$$-\frac{1}{2}\left(\frac{1}{\tau_{m\mathbf{k}}} |\phi_{m\mathbf{k}}\rangle\langle\phi_{n\mathbf{k}}| + \frac{1}{\tau_{n\mathbf{k}}} |\phi_{m\mathbf{k}}\rangle\langle\phi_{n\mathbf{k}}| \right).$$

The inverse of the dephasing time for the polarisation $T_{2,mn}^{\boldsymbol{k}}$ between two bands m and n is hence calculated as the average of the inverse scattering times $\tau_m^{\boldsymbol{k}}$, i.e. scattering rates, retrieved from the electron-phonon scattering calculations:

$$\frac{1}{T_{2,mn}^{\boldsymbol{k}}} = \frac{1}{2}\left(\frac{1}{\tau_m^{\boldsymbol{k}}} + \frac{1}{\tau_n^{\boldsymbol{k}}} \right).$$

Supplementary Fig. 8 shows the corresponding dephasing times affecting the polarisation between different bands.

The system of the SBEs is obtained by plugging the Bloch ansatz $\sum_{m,\boldsymbol{k}\in \mathrm{BZ}} a_m^{\boldsymbol{k}}(t)\, \varphi_m^{\boldsymbol{k}}(r)$ into the minimal coupling Hamiltonian $\mathcal{H}$ in length gauge and solving the equations for the time-dependent coefficients $a_m^{\boldsymbol{k}}(t)$, where $\varphi_m^{\boldsymbol{k}}$ is the Bloch function for band index m, with the associated energy band $\mathcal{H}_0 \varphi_m^{\boldsymbol{k}} = \varepsilon_m^{\boldsymbol{k}} \varphi_m^{\boldsymbol{k}}$. If we denote the products of two coefficients as $\rho_{mn}^{\boldsymbol{k}} = a_m^{\boldsymbol{k}}(t) a_n^{\boldsymbol{k}*}(t)$, the equations read

$$i\dot{\rho}_{mn}^{\boldsymbol{k}}(t) = \left(\varepsilon_{m}^{\boldsymbol{k}} - \varepsilon_{n}^{\boldsymbol{k}} - \frac{1-\delta_{mn}}{T_{2,mn}^{\boldsymbol{k}}}\right)\rho_{mn}^{\boldsymbol{k}}(t)$$

$$+E(t)\cdot\sum_{l}\left[D_{ml}^{\boldsymbol{k}}\rho_{ln}^{\boldsymbol{k}}(t) - D_{ln}^{\boldsymbol{k}}\rho_{ml}^{\boldsymbol{k}}(t)\right]$$

$$+iE(t)\cdot\nabla_{\boldsymbol{k}}\rho_{mn}^{\boldsymbol{k}}(t),$$

where $E(t)$ is the electrical field and $D_{mn}^{\boldsymbol{k}}$ are the transition dipole elements for $m \neq n$ and the Berry connections for $m = n$. They are calculated by $\boldsymbol{D}_{mn}^{\boldsymbol{k}} = i\langle\varphi_{m}^{\boldsymbol{k}}|\nabla_{\boldsymbol{k}}|\varphi_{n}^{\boldsymbol{k}}\rangle$ with the scalar product taken with respect to the unit cell. The last term in the equations, which couples the different k-points, can be omitted by transforming into a comoving frame and introducing a time-dependent $\boldsymbol{k} = \boldsymbol{K}(t) + \boldsymbol{A}(t)$. In that case, the quantity $\boldsymbol{D}_{mn}^{\boldsymbol{k}} \rightarrow \boldsymbol{D}_{mn}^{\boldsymbol{K}+\boldsymbol{A}(\boldsymbol{t})}$ becomes time-dependent and is shifted, where the magnitude of the shift is given by the vector potential $\boldsymbol{A}(t)$. The first line contains the dephasing time $T_{2,mn}^{\boldsymbol{k}}$ which depends on the band index m and momentum k.

The band structures and dipole elements were calculated using a tight binding model developed in [13]. In these calculations, we included spin-orbit coupling resulting in fully non-degenerate bands. The simulations were converged with a maximal time step of $\Delta t = 0.2$ a.u. and a Brillouin zone sampling of 300 × 300 points. The maximal field amplitude was set to $E_0 = 1.67 \cdot 10^{-3}$ a.u.

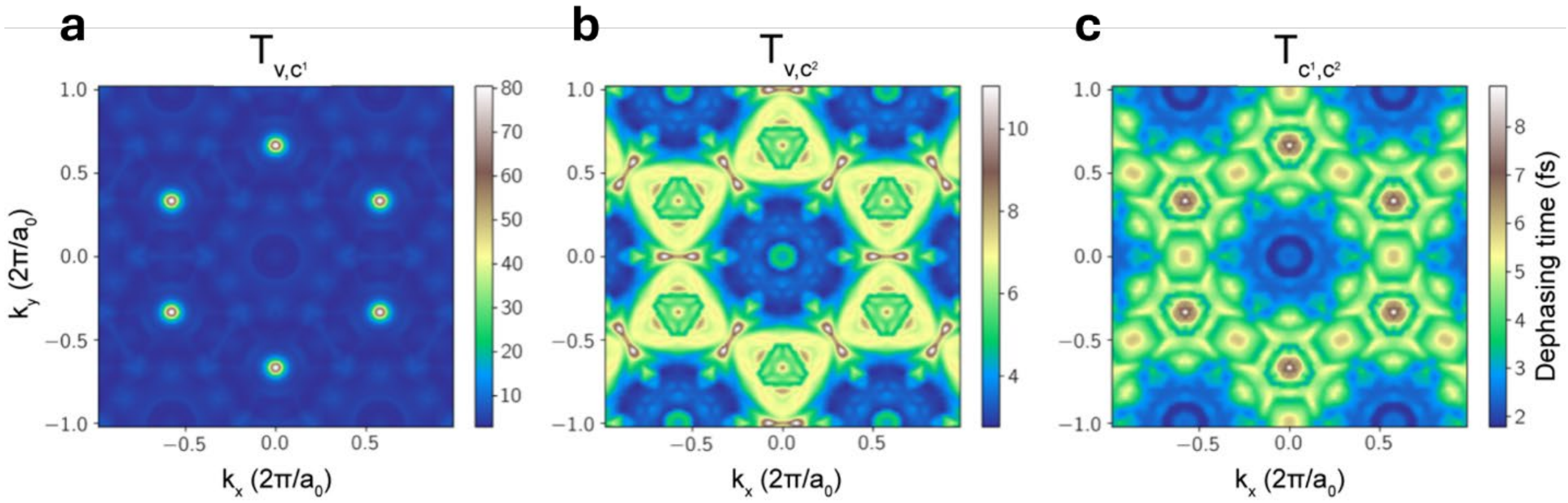

Supplementary Fig. 8. k-dependent dephasing times used in the SBE simulations for the dephasing between a) the valence and the first conduction band b) the valence and the second conduction band c) the two conduction bands.

<u>Supplementary references</u>

[1] Frisenda, R. *et al.* Recent progress in the assembly of nanodevices and van der Waals heterostructures by deterministic placement of 2D materials. *Chem. Soc. Rev.* **47**, 53–68 (2018).

[2] Mouri, S., Miyauchi, Y. & Matsuda, K. Tunable Photoluminescence of Monolayer MoS_2 via Chemical Doping. *Nano Lett* **13**, 5944–5948 (2013).

[3] Plechinger, G. *et al.* Identification of excitons, trions and biexcitons in single-layer WS_2. *physica status solidi (RRL) - Rapid Research Letters* **9**, 457–461 (2015).

[4] A. Croy. Bending rigidities and universality of flexural modes in 2D crystals. *Journ. Phys. Mater*. **3**, 02LT03 (2020).

[5] Giustino, F., Cohen, M. L., Louie, S. G. Electron-phonon interaction using Wannier functions. *Phys. Rev. B* **76**, 165108 (2007).

[6] Poncé, S., Margine, E. R., Verdi, C., Giustino, F. EPW: Electron–phonon coupling, transport and superconducting properties using maximally localized Wannier functions. *Comput. Phys. Commun*. **209**, 116-133 (2016).

[7] Giannozzi, P., Baseggio, O., Bonfà, P., Brunato, D., Car, R., Carnimeo, I., Cavazzoni, C., de Gironcoli, S., Delugas, P., Ruffino, F. F., Ferretti, A., Marzari, N., Timrov, I., Urru, A., Baroni, S. QUANTUM ESPRESSO toward the exascale. *Jorn. Chem. Phys.* **152**, 154105 (2020); http://www.quantum-espresso.org

[8] Mahatha, S. K., Ngankeu, A. S., Frank Hinsche, N., Mertig, I., Guilloy, K., Matzen, P. L., Bianchi, M., Sanders, C. E., Miwa, J. A., Bana, H., Travaglia, E., Lacovig, P., Bignardi, L., Lizzit, D., Larciprete, R., Baraldi, A., Lizzit, S., Hofmann, P. Electron–phonon coupling in single-layer MoS_2. *Surf. Sci.* **681**, 64-69 (2019).

[9] Yue, L., Gaarde, M. B., Introduction to theory of high-harmonic generation in solids: tutorial. *J. Phys. B* **39**, 535-555 (2022).

[10] Zhou, J.-J.; Park, J.; Lu, I.-T.; Maliyov, I.; Tong, X.; Bernardi, M. Perturbo: A Software Package for *Ab Initio* Electron–Phonon Interactions, Charge Transport and Ultrafast Dynamics. *Comp. Phys. Commun.* **264**, 107970 (2021).

[11] T. Lettau, H. A. M Leymann & J. Wiersig. Pitfalls in the theory of carrier dynamics in semiconductor quantum dots: Single-particle basis versus the many-particle configuration basis. *Phys. Rev. B* **95**, 085314 (2017).

[12] H.-P. Breuer and F. Petruccione. The Theory of Open Quantum Systems. 1st ed. Oxford University PressOxford, 2007. doi: 10.1093/acprof:oso/9780199213900.
001.0001.

[13] Liu, G.-B., Shan, W.-Y., Yao, Y., Yao, W., Xiao, D. Three-band tight-binding model for monolayers of group-VIB transition metal dichalcogenides. *Phys. Rev. B* **88**, 085433 (2013).
