# Supplementary material for: Strong field spectroscopy of many-body interactions in solids
Source: Nat Commun. 2026 Oct 20;17:10954. doi: 10.1038/s41467-026-78716-1 (PMC13680610; doi:10.1038/s41467-026-78716-1)
Supplement: Supplementary file 2 [file 41467_2026_78716_MOESM2_ESM.pdf]

Title: Supplementary Data 1

Description: The Supplementary Data 1 file consists of 6 Sheets containing the following data:

List 1 – Wavelength dependence of integral harmonic yield on the laser wavelength in WS2, armchair polarization orientation, Fig.2a, lower panel. Column A – laser wavelength in nm, Column B – normalized integral harmonic yield;

List 2 - Wavelength dependence of integral harmonic yield on the laser wavelength in WS2, zigzag polarization orientation, Fig.2a, upper panel. Column A – laser wavelength in nm, Column B – normalized integral harmonic yield;

List 3 - Wavelength dependence of integral harmonic yield on the laser wavelength in MoS2, armchair polarization orientation, Fig.2b, middle panel. Column A – laser wavelength in nm, Column B – normalized integral harmonic yield;

List 4 - Wavelength dependence of integral harmonic yield on the laser wavelength in MoS2, zigzag polarization orientation, Fig.2b, upper panel. Column A – laser wavelength in nm, Column B – normalized integral harmonic yield;

List 5 - Wavelength dependence of integral harmonic yield on the laser wavelength in CdSe, Fig.2b, lower panel. Column A – laser wavelength in nm, Column B – normalized integral harmonic yield;

List 6 – Dependence of the power index in the wavelength-dependent decay of the harmonic yield on the driving laser intensity in WS2 armchair polarization orientation, Fig.2c left panel, Column A – Laser intensity in TW/cm2, Column B – power index, Column C – Fit SE for Column C, and in WS2 zigzag polarization orientation, Fig.2c right panel, Column E – Laser intensity in TW/cm2, Column F – power index, Column G – Fit SE for Column C
