# Supplementary material for: Strong field spectroscopy of many-body interactions in solids
Source: Nat Commun. 2026 Oct 20;17:10954. doi: 10.1038/s41467-026-78716-1 (PMC13680610; doi:10.1038/s41467-026-78716-1)
Supplement: Supplementary file 3 [file 41467_2026_78716_MOESM4_ESM.pdf]

nature portfolio

Peer Review File

Strong Field Spectroscopy of Many-Body Interactions in Solids

Corresponding Author: Dr Daniil Kartashov

This file contains all reviewer reports in order by version, followed by all author rebuttals in order by version.

Version 0:

Reviewer comments:

Reviewer #1

(Remarks to the Author)
The manuscript presents a study of wavelength-dependent high-harmonic generation (HHG) in solids, incorporating momentum-dependent dephasing times derived from microscopic scattering calculations. The work addresses an important and timely question: how decoherence shapes strong-field electron dynamics and ultimately the scaling of HHG yield with driving wavelength. Overall, I find the manuscript interesting and potentially impactful. In particular, the introduction of k-resolved dephasing times is a valuable step toward a more realistic description of ultrafast dynamics in solids. The connection to experimentally observed wavelength scaling laws is also compelling. However, in its current form, I find that the central claim is not yet fully convincing, and several important aspects require clarification or additional analysis.
Major comments
1. Role and necessity of momentum-dependent dephasing The central advance of the paper is the use of a k-dependent dephasing time. While this is physically well motivated, the manuscript does not convincingly demonstrate that this level of complexity is required to explain the experimental observations. The key observable,the wavelength scaling of the HHG yield , has been studied extensively in prior work, and is known to be highly sensitive to the assumed dephasing time. The manuscript shows that with no dephasing → too weak scaling and very long dephasing → incorrect behavior
However, the crucial missing comparison is: How do the results change if one uses a single constant (phenomenological) dephasing time such as used in previous SBE calculations? Of course, I agree with the authors that there is a distinct behaviour for fixed T2 and T2(k), but are the experimental data sufficient to fully reproduce T2(k) and to distinguish different scattering mechanisms?
This is essential because: A constant T_2 can often be tuned to reproduce experimental scaling laws. Without this benchmark, it is unclear whether: the k-dependent dephasing is necessary, or it merely provides a more microscopic interpretation of an already effective phenomenology.
Recommendation:
Include a control calculation with a best-fit constant dephasing time, and directly compare:
wavelength scaling exponent
spectral shape
cutoff behavior

2. What is the effective dephasing time?
While the model introduces a k-dependent dephasing rate, it remains unclear what the effective coherence time is in practice. From a physical perspective, one would like to understand:
What is the typical range of T_2 (k) across the Brillouin zone?
What is the effective (trajectory-averaged) dephasing time relevant for HHG?
Right now, the manuscript lacks a clear physical takeaway. The reader is left without an intuitive understanding of “what the dephasing time actually is” in the regime relevant for HHG. I would also expect that this number depends on the harmonic order, the amount of additional charge carriers, and the temperature of the system. While it is of course more precise to include all these parameters, similar to what was mentioned above, I wonder if the measurement is sensitive to distinguish these effects as the experiment averages over all k-values, i.e., not only a single electron is driven.
Recommendation:
Provide:
a map of T_2 (k)
an effective averaged T_2 (e.g., weighted by emission strength)

comparison to experimentally inferred coherence times from prior literature

3. Consistency of the dephasing model with strong-field conditions
The dephasing rates appear to be derived from perturbative (field-free or weak-field) scattering calculations, while the HHG process itself occurs in a strong-field, highly non-equilibrium regime.
This raises an important conceptual concern: Under strong-field excitation carrier distributions are highly non-thermal, Floquet states or dressed bands may form (i.e., the bandstructure becomes modified), scattering channels may be modified Therefore it is unclear whether the employed dephasing rates remain valid in the regime where HHG is generated.
Recommendation. At minimum, discuss:
limitations of using field-free scattering rates
possible modifications under strong driving
whether this could systematically bias the results
4. Relation to prior work on HHG scaling and dephasing
The manuscript would benefit from a more complete discussion of prior literature: Wavelength scaling of HHG in solids has been reported previously, including strong dependence on coherence time. There are also earlier studies extracting or modeling dephasing times in solids from: HHG, four-wave mixing, time-resolved spectroscopy
Recommendation:
Expand the discussion and include references to:
prior wavelength-scaling studies
works addressing coherence and dephasing in HHG
comparisons to experimentally inferred T_2 values
Disucss scattering in intra- and interband electron dynamics

5. Interpretation of wavelength scaling
The manuscript attributes the observed scaling primarily to dephasing effects. While this is plausible, the interpretation may be somewhat overstated. Other factors that can influence scaling include:
trajectory length in k-space
band structure and dispersion
interband vs intraband contributions
dynamical Bloch oscillations
It would strengthen the paper to clarify: To what extent is the observed scaling uniquely determined by dephasing, versus other competing mechanisms?
Conclusion
In summary, this is a strong and interesting manuscript that addresses an important problem in ultrafast solid-state physics. The introduction of momentum-dependent dephasing is promising and could represent a meaningful advance.
However, the central claim would be significantly strengthened by:
A direct comparison to a constant dephasing model
A clearer physical interpretation of the effective dephasing time
A more careful discussion of the validity of the scattering model in strong fields
Addressing these points would make the conclusions much more compelling and the work more broadly impactful.

Reviewer #2

(Remarks to the Author)
Reviewer Comments
The manuscript presents an interesting combination of strong-field high-harmonic generation (HHG) experiments and first-principles calculations of electron–phonon scattering. The authors argue that momentum-resolved electron–phonon scattering dominates ultrafast decoherence in their experiment and use ab initio scattering rates to model the dephasing time T2 in semiconductor Bloch equation (SBE) simulations.
The work may be of interest to the strong-field solid-state community. However, several conceptual and technical points require clarification before the conclusions can be fully supported.
1. Novelty in relation to previous photo-doping studies
The interpretation proposed in the manuscript appears closely related to earlier photo-doping experiments and simulations, where the HHG yield was shown to depend strongly on the carrier density and the associated scattering processes.
For example, the work reported in the following study:
https://doi.org/10.1073/pnas.1706466114
demonstrates that the variation of HHG intensity with carrier density can be understood as the interplay of carrier–carrier and electron–phonon scattering. In that work, the authors also introduced a phenomenological relation in which the scattering rate (either carrier–carrier or carrier–phonon) is proportional to the inverse dephasing time 1/T2 within the SBE framework. Similar physical diagrams were used to interpret the scattering processes (see Equation 1 in that work for details).
In this context, the statement in the present manuscript that
“such new theory of ultrafast strong-field e–ph scattering needs to be developed, and the approach used in our work is currently the only feasible one”
appears somewhat strong and may require further justification.
From the perspective of the photo-doping literature, the results presented here may correspond to a regime where the carrier density remains relatively low, such that carrier–phonon scattering dominates over carrier–carrier scattering. This interpretation would be consistent with earlier studies. However, it is not immediately clear whether this conclusion is

expected to be universal.
In particular, when the driving field strength increases, the generated carrier density may grow significantly, and carrier–carrier scattering could become more important. At minimum, the possibility of such a regime should be discussed.
It would therefore be helpful if the authors could clarify more explicitly:
how the present results relate to the existing photo-doping literature; whether the observed dominance of electron–phonon scattering over carrier-carrier scattering is expected to be universal, or rather specific to the experimental regime (in particular the peak laser intensity) considered here.
Additional simulations performed at different laser intensities may help clarify this point.

2. Relation between the calculated scattering rate and the SBE dephasing time T2
I do not question the internal consistency of the calculations presented in the manuscript, nor the validity of the electron–phonon scattering model under the experimental parameters considered. I also fully agree that introducing a dephasing time T2 within the SBE framework is a well-established approach in modeling solid-state HHG. In addition, several previous works have already employed momentum-dependent dephasing times T2(k), for example:
Nature 533, 225 (2016)
Phys. Rev. Lett. 120, 253201(2018)
APL Computational Physics 1, 026104 (2025)
However, a conceptual point that may be important for many readers concerns the relation between the calculated scattering rate and the phenomenological dephasing time used in SBEs.
If I understand correctly, the derivation of 1/tau_n in Supplementary Section S4 likely relies on a Markov approximation, assuming that the bath correlation time is much shorter than the characteristic evolution time of the interband polarization. Under such conditions (which appear consistent with the laser parameters used in the manuscript), starting from a quantum master equation one typically obtains transition rates between bands that resemble Fermi's golden rule and involve scattering matrix elements.
The question then arises whether the resulting scattering rate can be directly identified with the 1/T2 used in the semiconductor Bloch equations.
In many HHG simulations, T2 is introduced as a phenomenological parameter describing the decay of the interband polarization. Therefore, the direct correspondence between the microscopic scattering rate and the effective SBE dephasing time is not always obvious.
It would significantly strengthen the manuscript if the authors could clarify:
under what assumptions the calculated scattering rate can be mapped onto the SBE dephasing time T2; whether additional contributions (for example pure dephasing or other many-body effects) might modify this correspondence.
A deeper mathematical and physical discussion addressing this point would help avoid potential misunderstandings.

3. Technical details
Some technical details regarding the experimental observable would benefit from clarification.
For example:
How exactly is the HHG yield defined in the experiment?
Over what photon-energy range or harmonic orders is the signal integrated?
Providing a more precise definition of the measured HHG yield would make the comparison between theory and experiment clearer.

Overall, the manuscript addresses an interesting problem in strong-field solid-state physics. Clarifying the points above would help better position the work within the existing literature and strengthen the physical interpretation of the results.

Version 1:

Reviewer comments:

Reviewer #1

(Remarks to the Author)
The reviewers have addressed most of my comments. In particular the extended discussion around T2 and its dependence on the k-value makes it more comprehensive. I can recommend publication in Nature Com.

Reviewer #2

(Remarks to the Author)
Overall Recommendation
I recommend acceptance of this manuscript for publication in its revised form. The authors have addressed my previous concerns thoroughly and thoughtfully, and the revised manuscript is substantially stronger in both its scientific presentation and its conceptual clarity.

The authors have responded to my previous comments with a careful and comprehensive revision. I particularly appreciate:

Clarification of the relation to prior photo-doping work. The authors have explicitly acknowledged previous studies demonstrating the sensitivity of HHG to carrier density and photodoping, and they now clearly state that the dominance of

electron-phonon scattering observed in their experiments is regime-specific rather than universal. The revised Discussion appropriately notes that carrier-carrier scattering may become important at substantially higher excited-carrier densities or under deliberate photodoping. This contextualization is valuable and appropriately positions the present work within the existing literature.

Explicit discussion of the mapping between microscopic scattering rates and the SBE dephasing time T2. The authors have added a clear explanation of the assumptions underlying their effective Markovian treatment, including the justification for treating scattering primarily as polarization dephasing rather than as population redistribution. The revised Supplementary Information provides the mathematical motivation in a Lindblad-type framework, and the authors have appropriately included caveats regarding pure dephasing, field dressing, memory effects, and other many-body contributions that may modify the effective dephasing time. This addresses my concern that the direct correspondence between the calculated scattering rate and the phenomenological SBE dephasing time should be made explicit.

Precise definition of the experimental HHG yield. The authors have now clearly defined the HHG yield in both the Results and Methods sections, specifying the spectral integration window (photon energies above the electronic bandgap within the experimentally accessible range of 200–1100 nm) and the correction procedures applied. This removes any ambiguity regarding the comparison between experiment and theory.

The authors' responses to the other reviewers' comments have also improved the manuscript, particularly the addition of the benchmark against constant-dephasing models, the trajectory-resolved effective dephasing analysis, and the clearer discussion of the limitations of field-free scattering rates under strong-field conditions.

Minor Technical Issue
While reviewing the revised manuscript and the supplementary materials, I noticed the following point that the authors should check:

The manuscript refers to "Supplementary Section 5" in the main text (appearing in the Results section where the authors state: "...conducted (i) ab-initio real-time time-dependent density functional (rt-TDDFT) calculations... and (ii) numerically solved the two-dimensional semiconductor Bloch equations (SBE) with infinite dephasing time (see Methods and Supplementary Section 5 for details)"). However, upon inspection of the supplementary materials provided with the original submission, I could only identify Sections 1–4. There does not appear to be a Section 5.

This is likely a minor oversight—perhaps the numbering was changed during revision, or the section was inadvertently omitted. I recommend that the authors verify whether Section 5 exists in the final supplementary file and, if not, correct the reference accordingly (e.g., to the appropriate section where the SBE details are presented, which appears to be Section 4 in the current supplementary materials).

Final Remarks
The manuscript presents a significant advance in our understanding of ultrafast dephasing in solid-state high-order harmonic generation. The authors have convincingly demonstrated that momentum-resolved electron-phonon scattering provides a microscopic origin for the few-femtosecond effective dephasing times required in SBE simulations of HHG. The combination of wavelength-dependent HHG measurements with first-principles calculations of scattering rates and strong-field simulations is compelling, and the revised manuscript now presents this work with appropriate nuance and clarity.

I congratulate the authors on a thorough and insightful study, and I recommend publication pending correction of the minor reference issue noted above.

Open Access This Peer Review File is licensed under a Creative Commons Attribution 4.0 International License, which permits use, sharing, adaptation, distribution and reproduction in any medium or format, as long as you give appropriate credit to the original author(s) and the source, provide a link to the Creative Commons license, and indicate if changes were made.
In cases where reviewers are anonymous, credit should be given to 'Anonymous Referee' and the source.
The images or other third party material in this Peer Review File are included in the article's Creative Commons license, unless indicated otherwise in a credit line to the material. If material is not included in the article's Creative Commons license and your intended use is not permitted by statutory regulation or exceeds the permitted use, you will need to obtain permission directly from the copyright holder.
To view a copy of this license, visit https://creativecommons.org/licenses/by/4.0/

Point-by-Point Response to the Reviewers

We thank the editor and the reviewers for their careful and constructive assessment of our manuscript. We are grateful that both reviewers found the work interesting and potentially impactful. The reports helped us clarify the central message of the paper and clarify several conceptual points, improving the presentation of the relation between microscopic electron-phonon scattering, effective dephasing in semiconductor Bloch equations, and the measured wavelength scaling of high-harmonic generation. Addressing reviewers comments and critical points, we have revised the manuscript substantially. In particular, we have added a main-text benchmark against constant-dephasing calculations, included a trajectory-resolved effective dephasing analysis, expanded the discussion of prior photo-doping and dephasing literature, clarified the limitations of field-free scattering rates in strong-field conditions, moderated the interpretation of wavelength scaling, and defined the experimental HHG yield more explicitly.

Reviewer #1

Comment 1

1. Role and necessity of momentum-dependent dephasing The central advance of the paper is the use of a k-dependent dephasing time. While this is physically well motivated, the manuscript does not convincingly demonstrate that this level of complexity is required to explain the experimental observations. The key observable, the wavelength scaling of the HHG yield , has been studied extensively in prior work, and is known to be highly sensitive to the assumed dephasing time. The manuscript shows that with no dephasing → too weak scaling and very long dephasing → incorrect behavior

However, the crucial missing comparison is: How do the results change if one uses a single constant (phenomenological) dephasing time such as used in previous SBE calculations? Of course, I agree with the authors that there is a distinct behavior for fixed T2 and T2(k), but are the experimental data sufficient to fully reproduce T2(k) and to distinguish different scattering mechanisms?

This is essential because: A constant T_2 can often be tuned to reproduce experimental scaling laws. Without this benchmark, it is unclear whether: the k-dependent dephasing is necessary, or it merely provides a more microscopic interpretation of an already effective phenomenology.

Recommendation:

Include a control calculation with a best-fit constant dephasing time, and directly compare:

wavelength scaling exponent

spectral shape

cutoff behavior.

We thank the reviewer for this important suggestion. We agree that the manuscript needed a direct benchmark against the commonly used phenomenological constant-dephasing approximation. In the revised manuscript, we have therefore added a new main-text section, "Benchmark against no dephasing or constant dephasing models," together with the new Fig. 3.

This new figure and text compare the experimentally retrieved wavelength-scaling exponents with SBE simulations using a momentum-independent constant T2. We find, consistent with previous work, that the retrieved scaling exponent is highly sensitive to the assumed constant dephasing time. A sufficiently tuned constant T2 can reproduce selected observables phenomenologically. However, such a value has to be chosen empirically and would have to be adjusted for different materials, crystal orientations, and excitation conditions. It therefore does not explain why an ultrashort dephasing time is required, nor does it identify the microscopic scattering mechanism responsible for it.

In addition, the final revised manuscript now includes a direct comparison of spectral shape and cutoff behavior in Fig. 3d. There, we compare spectra calculated with the best-to-experiment constant dephasing value, T2 = 5 fs, and with the k-dependent T2(k) model. The cutoff position is roughly similar in both descriptions, but the short constant dephasing produces strong suppression of the plateau structure at long driving wavelengths. This behavior is not observed in the experiment, where the plateau harmonics remain comparable to, or stronger than, the below-bandgap fifth harmonic. This comparison further supports the conclusion that the constant-T2 model can be used as a phenomenological benchmark but does not provide a complete physical description of the observed HHG response.

By contrast, the momentum-dependent T2(k) used in the revised manuscript follows from the calculated electron-phonon scattering landscape and reproduces the observed scaling without introducing an independently fitted dephasing time for each condition. We have revised the text to make this point more balanced: the experimental data should not be interpreted as a direct inversion of the full T2(k) landscape. Rather, the k-dependent model provides a microscopic explanation for the effective few-femtosecond dephasing that otherwise appears only as an empirical constant in phenomenological SBE calculations.

Comment 2

What is the effective dephasing time? While the model introduces a k-dependent dephasing rate, it remains unclear what the effective coherence time is in practice. From a physical perspective, one would like to understand:

What is the typical range of T_2 (k) across the Brillouin zone?

What is the effective (trajectory-averaged) dephasing time relevant for HHG?

Right now, the manuscript lacks a clear physical takeaway. The reader is left without an intuitive understanding of “what the dephasing time actually is” in the regime relevant for HHG. I would also expect that this number depends on the harmonic order, the amount of additional charge carriers, and the temperature of the system. While it is of course more

precise to include all these parameters, similar to what was mentioned above, I wonder if the measurement is sensitive to distinguish these effects as the experiment averages over all k-values, i.e., not only a single electron is driven.

Recommendation:

Provide:

a map of T_2 (k)

an effective averaged T_2 (e.g., weighted by emission strength)

comparison to experimentally inferred coherence times from prior literature

We agree with the reviewer that the original manuscript did not provide a sufficiently intuitive answer to the question "what is the effective dephasing time?" In the revised manuscript, we have therefore added a clearer physical discussion of the range of T2(k) and a new trajectory-resolved analysis.

The revised Fig. 4 now shows the 2D map of the momentum-dependent polarization dephasing time between the valence and first conduction bands. The revised text states that the dephasing time is relatively long near the valley, on the order of tens of femtoseconds, but drops below 10 fs once the carrier leaves the valley and reaches approximately 4 fs in parts of the Brillouin zone sampled by strong-field trajectories.

We also added the new Fig. 5, where representative electron trajectories for 3.5 and 4.5 μm driving wavelengths are overlaid with the dephasing-time landscape. From the dephasing profile along these trajectories, we calculate the trajectory-specific effective dephasing time. The resulting values are approximately 4 fs for the zigzag direction and 4.2-4.4 fs for the armchair direction, depending on the driving wavelength. This demonstrates explicitly how a few-femtosecond effective coherence time emerges even though the near-valley scattering time is much longer.

We also agree with the reviewer that the effective value can depend on harmonic order, carrier density, temperature, and the weighting of different trajectories in the measured ensemble. We have therefore avoided claiming that the experiment uniquely reconstructs all of these dependencies. Instead, the revised manuscript states that the HHG-relevant dephasing should be understood as a trajectory-dependent effective average over the momentum-space regions sampled by the driven carriers. We have also expanded the discussion of temperature and carrier-density dependence and cited prior work on coherence and dephasing times in solid-state optical measurements and HHG.

Comment 3

Consistency of the dephasing model with strong-field conditions

The dephasing rates appear to be derived from perturbative (field-free or weak-field) scattering calculations, while the HHG process itself occurs in a strong-field, highly non-equilibrium regime.

This raises an important conceptual concern: Under strong-field excitation carrier distributions are highly non-thermal, Floquet states or dressed bands may form (i.e., the band structure becomes modified), scattering channels may be modified

Therefore it is unclear whether the employed dephasing rates remain valid in the regime where HHG is generated.

Recommendation. At minimum, discuss:

limitations of using field-free scattering rates

possible modifications under strong driving

whether this could systematically bias the results

We thank the reviewer for raising this important limitation. We agree that the electron-phonon scattering rates used in the model are calculated in a field-free perturbative framework, whereas HHG occurs in a strongly driven and highly nonequilibrium regime.

We have revised the manuscript to state this limitation explicitly. The revised Results section now notes that under strong driving, dynamically dressed bands, nonthermal carrier distributions, and additional many-body channels may renormalize the effective scattering and dephasing rates. We also state that this may contribute to the remaining quantitative mismatch between the measured and simulated scaling exponents.

Accordingly, we now present the model as a first principles-informed effective description for the experimentally studied regime rather than as a complete theory of strong-field electron-phonon scattering. Its significance is that it captures the central physical trend: carriers driven away from the band extrema encounter much faster electron-phonon scattering, naturally producing a few-femtosecond effective dephasing time during HHG.

Comment 4

Relation to prior work on HHG scaling and dephasing

The manuscript would benefit from a more complete discussion of prior literature: Wavelength scaling of HHG in solids has been reported previously, including strong dependence on coherence time. There are also earlier studies extracting or modeling dephasing times in solids from: HHG, four-wave mixing, time-resolved spectroscopy

Recommendation:

Expand the discussion and include references to:

prior wavelength-scaling studies

works addressing coherence and dephasing in HHG

comparisons to experimentally inferred T_2 values

Discuss scattering in intra- and interband electron dynamics

We agree that the relation to previous work should be clearer. In the revised manuscript, we have expanded the Introduction and Discussion and added references to prior studies of HHG wavelength scaling, phenomenological dephasing in solid-state HHG simulations, electron-hole coherence in strongly driven two-dimensional materials, weak-field coherence times, and photo-doping-induced scattering effects.

The revised Introduction now states that previous studies have shown the HHG response of solids to be highly sensitive to the assumed dephasing time and to carrier populations, including under photo-doping conditions. We also now discuss the difference between coherence times known from weak-field optical measurements near the band edge and the much shorter effective dephasing times required to model strong-field HHG.

In addition, the revised Methods and Supplementary Information clarify how scattering is included in the SBE model. In the present approximation, scattering is treated primarily as dephasing of the interband polarization rather than as explicit redistribution of carrier occupations. We now state this assumption more clearly and explain that a full treatment of carrier redistribution would require a kinetic Boltzmann approach beyond the present scope.

Comment 5

Interpretation of wavelength scaling

The manuscript attributes the observed scaling primarily to dephasing effects. While this is plausible, the interpretation may be somewhat overstated. Other factors that can influence scaling include:

trajectory length in k-space

band structure and dispersion

interband vs intraband contributions

dynamical Bloch oscillations

It would strengthen the paper to clarify: To what extent is the observed scaling uniquely determined by dephasing, versus other competing mechanisms?

Conclusion

In summary, this is a strong and interesting manuscript that addresses an important problem in ultrafast solid-state physics. The introduction of momentum-dependent dephasing is promising and could represent a meaningful advance.

However, the central claim would be significantly strengthened by:

A direct comparison to a constant dephasing model

A clearer physical interpretation of the effective dephasing time

A more careful discussion of the validity of the scattering model in strong fields

Addressing these points would make the conclusions much more compelling and the work more broadly impactful.

We agree that the wavelength scaling should not be presented as being uniquely determined by dephasing alone. In the revised manuscript, we have moderated this interpretation and explicitly state that the scaling also depends on the band structure, driven trajectory length in momentum space, and the relative interband and intraband contributions.

At the same time, our simulations show that, within a model that already includes realistic band structure, full two-dimensional Brillouin-zone dynamics, and multiple bands, the steepness of the experimentally observed scaling is most sensitively controlled by the dephasing term. Calculations without dephasing, or with long constant dephasing times, yield substantially weaker scaling. Introducing momentum-resolved dephasing derived from electron-phonon scattering rates reproduces the observed trend more consistently.

We have therefore revised the manuscript to present dephasing as the dominant contribution governing the scaling exponent under the present experimental conditions, while clearly stating that other ingredients shape the detailed spectral response. We believe this revision addresses the reviewer's concern and provides a more balanced interpretation.

Reviewer #2

Comment 1

Novelty in relation to previous photo-doping studies

The interpretation proposed in the manuscript appears closely related to earlier photo-doping experiments and simulations, where the HHG yield was shown to depend strongly on the carrier density and the associated scattering processes.

For example, the work reported in the following study:

https://doi.org/10.1073/pnas.1706466114

demonstrates that the variation of HHG intensity with carrier density can be understood as the interplay of carrier–carrier and electron–phonon scattering. In that work, the authors also introduced a phenomenological relation in which the scattering rate (either carrier–carrier or carrier–phonon) is proportional to the inverse dephasing time $1/T2$ within the SBE framework. Similar physical diagrams were used to interpret the scattering processes (see Equation 1 in that work for details).

In this context, the statement in the present manuscript that “such new theory of ultrafast strong-field e–ph scattering needs to be developed, and the approach used in our work is currently the only feasible one” appears somewhat strong and may require further justification.

From the perspective of the photo-doping literature, the results presented here may correspond to a regime where the carrier density remains relatively low, such that carrier–phonon scattering dominates over carrier–carrier scattering. This interpretation would be

consistent with earlier studies. However, it is not immediately clear whether this conclusion is expected to be universal.

In particular, when the driving field strength increases, the generated carrier density may grow significantly, and carrier–carrier scattering could become more important. At minimum, the possibility of such a regime should be discussed. It would therefore be helpful if the authors could clarify more explicitly: how the present results relate to the existing photo-doping literature; whether the observed dominance of electron–phonon scattering over carrier-carrier scattering is expected to be universal, or rather specific to the experimental regime (in particular the peak laser intensity) considered here.

Additional simulations performed at different laser intensities may help clarify this point.

We thank the reviewer for this important comment. We agree that the relation to previous photo-doping work and the regime dependence of the scattering mechanism should be stated more clearly. We have revised the manuscript accordingly.

The revised Introduction now acknowledges previous work showing that HHG in solids is sensitive to carrier population and photodoping. In the revised Results section, we explicitly discuss electron-electron scattering as a possible dephasing mechanism and compare its expected carrier-density sensitivity with our intensity-dependent measurements. Based on the Keldysh strong-field excitation rate, we estimate that the excited carrier density changes by up to approximately one order of magnitude over the investigated intensity range. If carrier-carrier scattering were the dominant contribution, this change would be expected to produce a pronounced change in the effective dephasing time and hence in the wavelength-scaling exponent. Experimentally, however, the exponent changes by less than 10% when the laser intensity is varied by more than 70%. We therefore conclude that carrier-carrier scattering is not the dominant contribution under the specific experimental conditions studied here. Importantly, we have revised the manuscript to avoid presenting this conclusion as universal. The revised Discussion now states that carrier-carrier scattering may become important at substantially higher excited-carrier densities, under deliberate photodoping, or in more strongly driven regimes. We have also removed the overly strong implication that the present field-free scattering treatment is "the only feasible" approach and replaced it with a more careful statement that it is a first principles-informed effective description for the present regime.

Comment 2

Relation between the calculated scattering rate and the SBE dephasing time T2

I do not question the internal consistency of the calculations presented in the manuscript, nor the validity of the electron–phonon scattering model under the experimental parameters considered. I also fully agree that introducing a dephasing time T2 within the SBE framework is a well-established approach in modeling solid-state HHG. In addition, several previous works have already employed momentum-dependent dephasing times T2(k), for example:

Nature 533, 225 (2016)

Phys. Rev. Lett. 120, 253201(2018)

APL Computational Physics 1, 026104 (2025)

However, a conceptual point that may be important for many readers concerns the relation between the calculated scattering rate and the phenomenological dephasing time used in SBEs.

If I understand correctly, the derivation of 1/tau_n in Supplementary Section S4 likely relies on a Markov approximation, assuming that the bath correlation time is much shorter than the characteristic evolution time of the interband polarization. Under such conditions (which appear consistent with the laser parameters used in the manuscript), starting from a quantum master equation one typically obtains transition rates between bands that resemble Fermi's golden rule and involve scattering matrix elements.

The question then arises whether the resulting scattering rate can be directly identified with the 1/T2 used in the semiconductor Bloch equations.

In many HHG simulations, T2 is introduced as a phenomenological parameter describing the decay of interband polarization. Therefore, the direct correspondence between the microscopic scattering rate and the effective SBE dephasing time is not always obvious.

It would significantly strengthen the manuscript if the authors could clarify:

under what assumptions the calculated scattering rate can be mapped onto the SBE dephasing time T2; whether additional contributions (for example pure dephasing or other many-body effects) might modify this correspondence.

A deeper mathematical and physical discussion addressing this point would help avoid potential misunderstandings.

We appreciate this comment. In the revised manuscript and Supplementary Information, we now explain the mapping between the microscopic electron-phonon scattering rates and the effective SBE dephasing time more explicitly.

In the revised Methods section, we state that the calculated band- and momentum-resolved electron-phonon scattering rates are applied to the interband polarization dynamics. Thus, in the present SBE treatment, scattering is treated as a source of polarization dephasing rather than explicitly as a redistribution of carrier occupations. This approximation is motivated by the fact that the HHG yield in the present regime is particularly sensitive to the coherence of the interband polarization, while a full kinetic treatment of population redistribution would require a more involved Boltzmann-equation approach.

The revised Supplementary Information provides the corresponding mathematical motivation in a Lindblad-type framework. In this effective Markovian description, scattering channels with rates obtained from the electron-phonon self-energy act as dissipative processes that dephase the microscopic polarization. The inverse dephasing time for the polarization between bands is therefore constructed from the relevant band-resolved scattering rates.

We have also added an explicit caveat that this mapping is not an exact identity in the most general many-body sense. Pure dephasing, carrier-carrier correlations, defect scattering, memory effects, field dressing, and other many-body contributions may modify effective dephasing time. We therefore present T2(k) as a physically motivated and first-principles-informed effective dephasing term for the present regime rather than as a universal exact correspondence.

Comment 3

Technical details

Some technical details regarding the experimental observable would benefit from clarification.

For example:

How exactly is the HHG yield defined in the experiment?

Over what photon-energy range or harmonic orders is the signal integrated?

Providing a more precise definition of the measured HHG yield would make the comparison between theory and experiment clearer.

Overall, the manuscript addresses an interesting problem in strong-field solid-state physics. Clarifying the points above would help better position the work within the existing literature and strengthen the physical interpretation of the results.

We agree that the experimental observable should be defined more explicitly. We have revised both the Results and Methods sections accordingly.

In the revised Results section, we now state that the HHG yield is defined as the detector- and grating-corrected spectral intensity, converted to the frequency domain and integrated over photon energies above the electronic bandgap within the experimentally accessible window shown by the shaded region in Fig. 1. We also state that the same integration window is used for comparison with numerical simulations.

The revised Methods section repeats this definition and adds the relevant experimental details. The harmonics were measured in the 200-1100 nm wavelength range, limited by the spectrometer and detector. The spectra were corrected for the wavelength-dependent response of the spectrometer and detector, converted to the frequency domain, and integrated above the material bandgap within the experimentally accessible spectral window. The resulting integrated quantity is referred to throughout the manuscript as the HHG yield.
